# PP2A phosphatase is required for dendrite pruning via actin regulation in *Drosophila*

**DOI:** 10.15252/embr.201948870

**Published:** 2020-03-24

**Authors:** Neele Wolterhoff, Ulrike Gigengack, Sebastian Rumpf

**Affiliations:** ^1^ Institute for Neurobiology University of Münster Münster Germany

**Keywords:** actin, dendrite, phosphatase, PP2A, pruning, Cell Adhesion, Polarity & Cytoskeleton, Neuroscience, Post-translational Modifications, Proteolysis & Proteomics

## Abstract

Large‐scale pruning, the developmentally regulated degeneration of axons or dendrites, is an important specificity mechanism during neuronal circuit formation. The peripheral sensory class IV dendritic arborization (c4da) neurons of *Drosophila* larvae specifically prune their dendrites at the onset of metamorphosis in an ecdysone‐dependent manner. Dendrite pruning requires local cytoskeleton remodeling, and the actin‐severing enzyme Mical is an important ecdysone target. In a screen for pruning factors, we identified the protein phosphatase 2 A (PP2A). PP2A interacts genetically with the actin‐severing enzymes Mical and cofilin as well as other actin regulators during pruning. Moreover, *Drosophila* cofilin undergoes a change in localization at the onset of metamorphosis indicative of a change in actin dynamics. This change is abolished both upon loss of Mical and PP2A. We conclude that PP2A regulates actin dynamics during dendrite pruning.

## Graphical abstract

The protein phosphatase PP2A is required for pruning, the regulated elimination of neurites during neuronal development, by promoting the disassembly of actin structures.

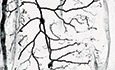

## Introduction

Neurite pruning, the physiological degeneration of axons or dendrites during development, is an important specificity mechanism during neuronal circuit formation 1, 2. While the mechanisms of neurite outgrowth and synapse formation have been studied in some detail, comparably little is known about the mechanisms underlying pruning. In holometabolous insects, pruning occurs at large scale during metamorphosis to remove larval‐specific neurites. In the peripheral nervous system (PNS) of *Drosophila*, the so‐called class IV dendritic arborization (c4da) neurons specifically prune their long and branched larval dendrites at the onset of the pupal phase by a degenerative mechanism 3, 4. C4da neuron dendrite pruning is induced by a prepupal peak in the steroid hormone ecdysone 3, 4. Ecdysone acts cell‐autonomously in c4da neurons by inducing the expression of pruning genes such as the transcription factor Sox14 and the actin‐severing enzyme Mical 5, an oxidoreductase that can sever actin filaments through actin oxidation 6, as well as the putative growth regulator headcase 7. The first morphological signs of dendrite pruning are seen at around 3–5 h after puparium formation (h APF), when dendrites start to show local thinnings and varicosities in their proximal regions that appear to be due to changes in cytoskeletal and membrane stability. At these proximal sites, the dendrites are eventually severed between 6 and 12 h APF. Known pathways that contribute to this destabilization of the proximal dendrites are local microtubule disassembly 4, 8, 9 and alterations in plasma membrane trafficking 10, 11, 12, 13. Microtubule disassembly is induced by a signal transduction cascade involving the kinase Par‐1 which negatively regulates the microtubule‐associated protein tau to enhance microtubule dynamics 9. Loss of microtubules starts at proximal dendrite branchpoints and proceeds with a proximal‐to‐distal directionality 14. Dendritic microtubules display a uniform plus end‐in orientation, and this uniformity is required for dendrite pruning 14. Together with the observed directionality of microtubule breakdown, this indicates that microtubule breakdown occurs mostly from plus ends during dendrite pruning and that microtubule polarity acts as a spatial determinant 15. Once severed, pruned dendrites are subsequently fragmented in a caspase‐dependent manner and phagocytosed by epidermal cells 16, 17.

While the roles and importance of microtubule regulation during pruning are emerging, relatively little is known about actin regulation during the process. While it is clear that the actin‐severing enzyme Mical is induced in c4da neurons at the onset of the pupal stage and is required for dendrite pruning 5, it is not known whether it affects actin structures at this stage, and whether other actin regulators are involved in dendrite pruning. This is somewhat surprising because actin dynamics play important roles during earlier larval c4da neuron dendrite development. For example, Arp2/3‐dependent actin polymerization promotes dendrite branching 18, and this also requires the actin disassembly factor cofilin to increase actin dynamics 19. One factor that has recently been implicated in actin regulation during larval c4da neuron dendrite development is Widerborst (Wdb), encoding a regulatory B subunit of protein phosphatase 2A (PP2A) 20. PP2A is known to regulate both microtubules, e.g., through tau 21, and actin, e.g., through Rho GTPases or regulators of cortical actin 22, 23. It is a trimeric protein complex consisting of an A subunit that acts as a scaffold (encoded by PP2A‐29B in flies), a C subunit containing the active site (encoded by *microtubule star*/mts), and one of several regulatory B subunits. In addition to Wdb, the B subunit Twins (Tws) has also been linked to actin regulation during imaginal disk development 24.

In this study, we identify PP2A as a regulator of c4da neuron dendrite pruning. In a survey of phosphoregulators of dendrite pruning, we found that loss of the scaffold subunit PP2A‐29B caused strong dendrite pruning defects. The catalytic subunit mts and—in a somewhat redundant manner, the B subunits Wdb and Tws are also required for this process, indicating the involvement of a specific PP2A holoenzyme. During dendrite pruning, PP2A displays genetic interactions with Mical, the actin disassembly factor cofilin and other actin regulators, indicating that PP2A acts via regulated actin disassembly during the process.

## Results

### The serine/threonine phosphatase PP2A is required for c4da neuron dendrite pruning

**Figure 1:**
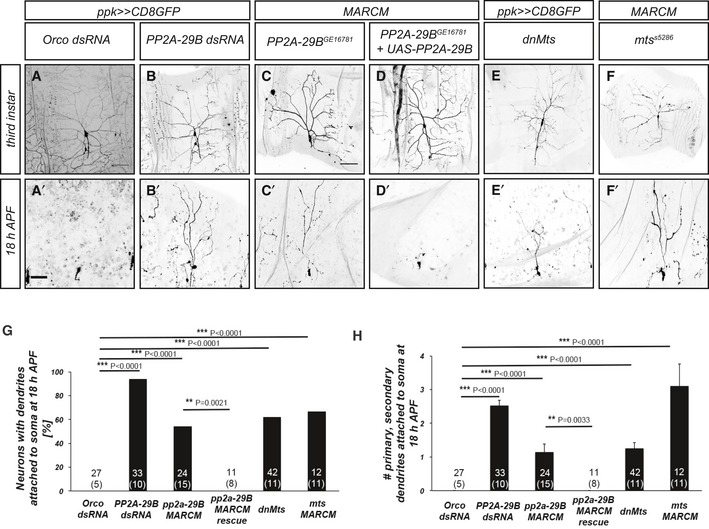
PP2A is required for dendrite pruning A–F’Upper panels (A–F) show morphology of c4da neurons of the indicated genotypes at the third‐instar larval stage, and lower panels (A’–F’) show c4da neuron morphology at 18 h after puparium formation (APF). Neurons were labeled by CD8::GFP expression under *ppk‐GAL4* or by tdTomato expression in MARCM clones. (A, A’) C4da neurons expressing control Orco dsRNA. Scale bars are 100 μm in (A) (for larval images) and 50 μm in (A’) (for pupal images). (B, B’) C4da neurons expressing PP2A‐29B dsRNA. (C, C’) Homozygous *PP2A‐29B* ^*GE16781*^ mutant c4da neuron MARCM clones. (D, D’) Homozygous *PP2A‐29B* ^*GE16781*^ mutant c4da neuron MARCM clones expressing UAS‐^HA^PP2A‐29B. (E, E’) C4da neurons expressing dominant‐negative Mts. (F, F’) Homozygous *mts* ^*s5286*^ mutant c4da neuron MARCM clones.GPenetrance of pruning defects at 18 h APF in (A’–E’). **P < 0.005, ***P < 0.0005, Fisher's exact test. The number of neurons for each genotype is given in the figure, and the number of animals is given below in parentheses.HSeverity of pruning defects at 18 h APF in (A’–E’) as assessed by number of primary and secondary dendrites attached to soma at 18 h APF. Data are mean ± s.d., **P < 0.005, ***P < 0.0005, Wilcoxon's test. Numbers of neurons (animals) for each genotype are given in the figure.

**Figure EV1:**
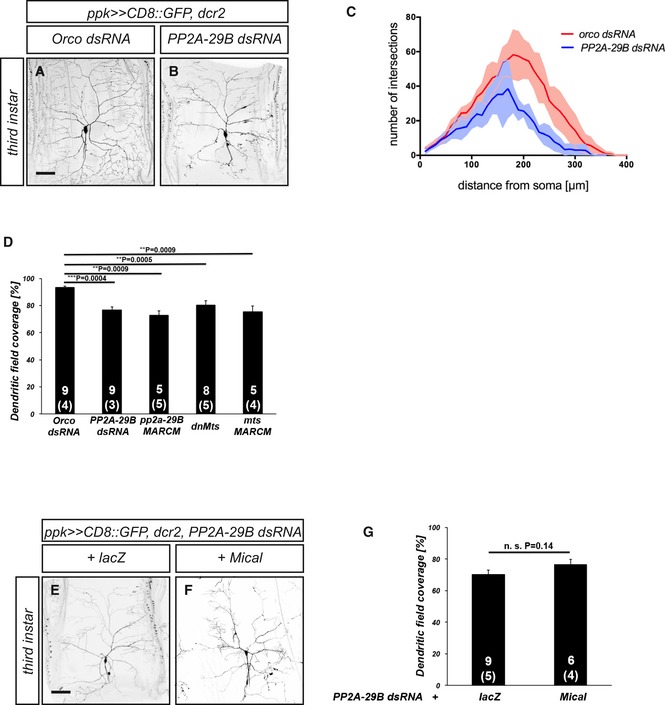
Effects of PP2A‐29B knockdown on larval dendrite morphology A, BMorphology of control c4da neurons (A) or c4da neurons expressing PP2A‐29B dsRNA under *ppk‐GAL4* (B) at the third‐instar larval stage. Scale bar is 100 μm.CSholl analysis of dendrite branch number and distribution for control c4da neurons and c4da neurons expressing PP2A‐29B dsRNA. N was 9 neurons (from 3 animals) for Orco dsRNA and 5 neurons (from 2 animals) for PP2A‐29B dsRNA, respectively.DDendritic field coverage of control c4da neurons and c4da neurons upon PP2A manipulation, respectively. Neurons for genotypes other than the Orco dsRNA control and PP2A‐29B dsRNA include those shown in Fig 1 C, E, F. The number of neurons (animals) in each sample is given in the graph. Data are mean ± s.d., and the *P* value was calculated using Wilcoxon's test.E, FMorphology of c4da neurons coexpressing PP2A‐29B and lacZ (E) or c4da neurons coexpressing PP2A‐29B and Mical (F) at the third‐instar larval stage. Scale bar is 100 μm.GDendritic field coverage of c4da neurons in (E and F). The number of neurons (animals) in each sample is given in the graph. Data are mean ± s.d.. n.s., not significant, Wilcoxon's test.

In order to identify phosphoregulators of c4da neuron dendrite pruning, we expressed dsRNA constructs targeting signaling molecules under the control of the c4da neuron‐specific GAL4 driver *ppk‐GAL4* and visualized c4da neurons by coexpression of membrane‐bound GFP. Control c4da neurons expressing *Orco* dsRNA (against *Odorant receptor co‐receptor* which is not expressed in c4da neurons) have long and branched dendrites at the third‐instar larval stage (Fig 1A), which are completely and specifically pruned at 18 h APF (Fig 1A’, G, and H). Loss of *PP2A‐29B*, encoding the A subunit of the serine/threonine phosphatase PP2A, or the catalytic C subunit encoded by *Microtubule star* (*mts*), caused a decrease in branching and dendritic field coverage at the third‐instar larval stage (Figs 1B–F and EV1). At 18 h APF, PP2A‐29B knockdown caused dendrite pruning defects (Fig 1B’). This phenotype was highly penetrant, as most c4da neurons showed pruning defects at this stage (Fig 1G), and also very severe, as most neurons retained many primary and secondary dendrites still attached to the soma (Fig 1H). In order to confirm this result, we next used Mosaic Analysis with a Repressible Cell Marker (MARCM) 25 to generate homozygous *PP2A‐29B* mutant c4da neurons. C4da neurons homozygous for the loss‐of‐function allele *PP2A‐29B* ^*GE16781*^ also displayed dendrite pruning defects at 18 h APF (Fig 1C’, G, and H). Furthermore, these pruning defects could be rescued by GAL4/UAS‐mediated expression of HA‐tagged PP2A‐29B in the MARCM clones (Fig 1D’, G, and H).

In order to assess the requirement for PP2A catalytic activity, we next expressed a dominant‐negative version of the catalytic subunit, dnMts, in c4da neurons. Similar to the effects of PP2A‐29B downregulation, the expression of dnMts caused strong and penetrant dendrite pruning defects at 18 h APF (Fig 1E, E’, G, and H). In order to confirm this result, we again used MARCM to create c4da neurons homozygous for *mts* ^*s5286*^, a P element insertion in the Mts gene. These c4da neurons also displayed strong dendrite pruning defects (Fig 1F–H).

**Figure EV2:**
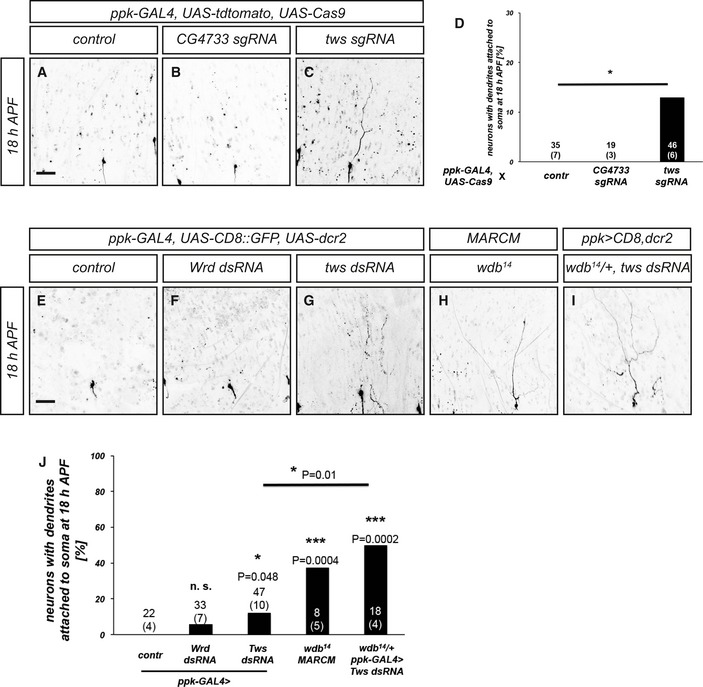
PP2A B subunits required for dendrite pruning A–CMorphology of c4da neurons expressing the indicated CRISPR/Cas9 constructs at 18 h after puparium formation (APF). C4da neurons were labeled by expression of tdTomato under *ppk‐GAL4*. (A) Control c4da neuron expressing UAS‐Cas9. (B) C4da neuron expressing UAS‐Cas9 and an sgRNA construct against PR72/CG4733. (C) C4da neuron expressing UAS‐Cas9 and an sgRNA construct against Twins/Tws.DPenetrance of pruning defects at 18 h APF in (A–C). Numbers of neurons (animals) for each genotype are given in the figure. *P < 0.05, Fisher's exact test.E–IMorphology of c4da neurons of the indicated genotypes at 18 h after puparium formation (APF). C4da neurons were labeled by the expression of CD8::GFP under *ppk‐GAL4*, or by tdTomato expression in MARCM clones. (E) Control c4da neuron not expressing a dsRNA construct. (F) C4da neuron expressing wrd dsRNA under *ppk‐GAL4*. (G) C4da neuron expressing tws dsRNA under *ppk‐GAL4*. (H) C4da neuron MARCM clone homozygous for the *wdb* ^*14*^ mutation. (I) C4da neuron expressing tws dsRNA under *ppk‐GAL4* in a *wdb* ^*14*^/+ heterozygous mutant background.JPenetrance of pruning defects at 18 h APF in (E–I). Numbers of neurons (animals) for each genotype are given in the figure. *P < 0.05, ***P < 0.0005, Fisher's exact test.Data information: The scale bars in (A and E) are 50 μm.

In order to test which B subunit might be involved in the regulation of c4da neuron dendrite pruning, we used sgRNAs or dsRNA constructs for tissue‐specific CRISPR and/or RNAi, as well as mutant analyses. The four B/B’ subunits in the fly genome are encoded by *Twins* (Tws), *PR72/CG4733, Well rounded* (Wrd), and *Widerborst* (Wdb). While the expression of an sgRNA construct directed against CG4733 under *ppk‐GAL4* did not cause dendrite pruning defects at 18 h APF, an sgRNA construct against *Tws* resulted in mild pruning defects (Fig EV2A–D). The expression of a dsRNA construct against Wrd did not cause significant dendrite pruning defects (Fig EV2F), while the expression of a dsRNA construct against Tws again caused very mild defects (Fig EV2G and J). In order to manipulate Widerborst, we used the mutant allele *wdb* ^*14*^ for MARCM analysis. Homozygous *wdb* ^*14*^ c4da neuron MARCM clones displayed clear dendrite pruning defects (Fig EV2H). However, the penetrance of these defects was still lower than expected from the very strong defects caused by manipulation of *PP2A‐29B* or *Mts*. PP2A B subunits can sometimes act in a redundant manner 26. To address this possibility, we expressed Tws dsRNA in c4da neurons in a *wdb* ^*14*^ */+* heterozygous mutant background. This manipulation caused stronger dendrite pruning defects more similar to those caused by loss of *PP2A‐29B* or Mts (Fig EV2I and J), indicating that these two B subunits can regulate pruning redundantly. Taken together, our data indicate that a PP2A holoenzyme complex is required for c4da neuron dendrite pruning.

### PP2A does not interact with the Par‐1/tau microtubule pathway during dendrite pruning

**Figure EV3:**
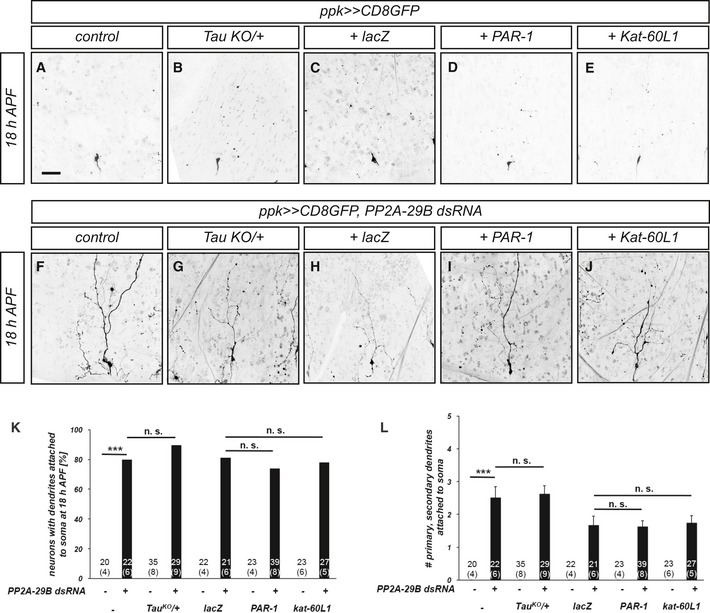
The role of PP2A during c4da neuron dendrite pruning is not linked to Par‐1‐mediated microtubule disassembly A–JMicrotubule pathway components were tested for their ability to suppress pruning defects induced by PP2A‐29B dsRNA expression. C4da neurons were labeled by CD8GFP expression under *ppk‐GAL4*, and dendrite pruning defects were assessed at 18 h APF. Panels (A–E) show effects of microtubule pathway manipulations alone, and panels (F–J) show neurons coexpressing PP2A‐29B dsRNA. Scale bar in A is 50 μm. (A, F) Control c4da neurons. (B, G) C4da neurons in Tau^KO^/+ heterozygous background. (C, H) C4da neurons (co‐)expressing UAS‐lacZ. (D, I) C4da neurons (co‐)expressing PAR‐1. (E, J) C4da neurons (co‐)expressing ^Venus^Kat‐60L1.KPenetrance of pruning defects in (A–J). ***P < 0.0005, n.s., not significant, Fisher's exact test. Numbers of analyzed neurons (animals) are given in the graph.LSeverity of pruning defects in (A–J) at 18 h APF as assessed by number of primary and secondary dendrites attached to soma at 18 h APF. Data are mean ± s.d., and numbers of analyzed neurons (animals) are given in the graph. ***P < 0.0005, Wilcoxon's test, n.s., not significant.

We next asked how PP2A could regulate dendrite pruning. Because the role of PP2A in cytoskeleton regulation is well established, we wanted to assess a potential role for PP2A in microtubule disassembly. To this end, we expressed PP2A‐29B dsRNA in c4da neurons and tested whether upregulation of the microtubule disassembly pathway(s) involved in dendrite pruning could ameliorate the dendrite pruning defects caused by this treatment. *Tau* heterozygosity or overexpression of Par‐1 or kat‐60L1 did not cause dendrite pruning defects by themselves (Fig EV3A–E). We next assessed whether these manipulations suppressed the pruning defects induced by PP2A downregulation. In order to control for potential UAS titration effects, we compared the effects of *PAR‐1* and *kat‐60L1* overexpression to overexpression of *lacZ*. However, none of the microtubule manipulations suppressed the pruning defects induced by loss of *PP2A‐29B* (Fig EV3F–J), suggesting that the function of PP2A during c4da neuron dendrite pruning is not linked to Par‐1/tau‐mediated microtubule regulation.

**Figure EV4:**
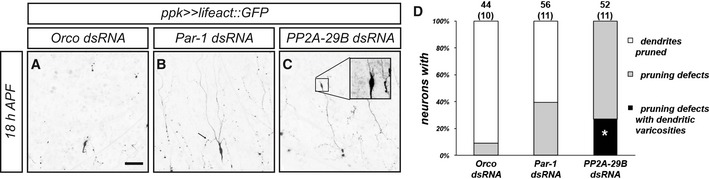
Loss of PP2A causes unusual dendritic varicosities A–DLoss of PP2A induces large distal varicosities in unpruned dendrites at 18 h APF. The F‐actin reporter lifeact::GFP was expressed under *ppk‐GAL4* in c4da neurons expressing the indicated dsRNA constructs and imaged at 18 h APF. (A) Orco dsRNA control. (B) Par‐1 dsRNA. The arrow denotes a small varicosity in a proximal dendrite. (C) PP2A‐29B dsRNA. The inset shows an enlarged image of a large varicosity at the tip of an unpruned dendrite. (D) Quantification of pruning defects and dendritic varicosities in (A–C). Categories are as follows: neurons with pruned dendrites (white), neurons with dendrite pruning defects (i.e., still attached to soma) (gray), and neurons with pruning defects and big distal varicosities (black). For the last category, we compared Par‐1 dsRNA with PP2A‐29B dsRNA. The number of neurons (and number of animals) for each genotype is given in each graph (data were not derived from independent experiments). *P < 0.05, Fisher's exact test.

The pruning defects caused by loss of PP2A also seemed qualitatively different from the defects caused by loss of PAR‐1. Pruning dendrites of control c4da neurons develop varicosities as a sign of degeneration during the early pruning stages between approximately 3 h APF and 12 h APF 10. These varicosities are round, occur in thinned regions of (proximal) dendrites, and are usually smaller than 5 μm in diameter. In contrast, unpruned c4da neuron dendrites expressing *PP2A‐29B* dsRNA displayed varicosities at 18 h APF that were larger and often occurred at distal dendrite tips. Sometimes, they developed small filopodia‐like protrusions (e.g., Fig EV4C), somewhat reminiscent of axonal growth cones, and these varicosities also were positive for the actin marker lifeact::GFP 27. In unpruned dendrites of c4da neurons lacking PAR‐1, only small, proximal varicosities could be seen that were reminiscent of the ones normally occurring in earlier (Fig EV4A–D, arrow in Fig EV4B).

### PP2A interacts genetically with ecdysone target genes

**Figure 2:**
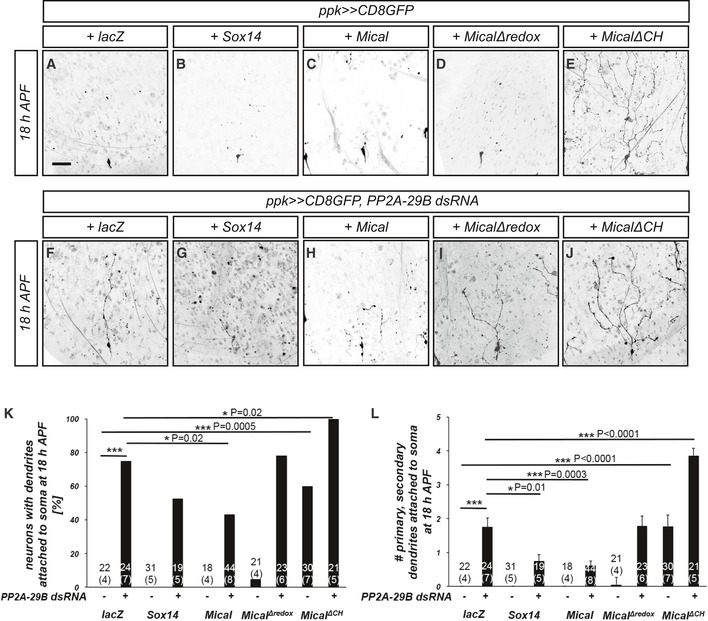
PP2A interacts genetically with the Sox14/Mical pathway A–JThe indicated Sox14 or Mical constructs were tested for their ability to suppress pruning defects induced by PP2A‐29B dsRNA expression. C4da neurons were labeled by CD8::GFP expression under *ppk‐GAL4*, and dendrite pruning defects were assessed at 18 h APF. Panels (A–E) show effects of Sox14/Mical pathway manipulations alone, and panels (F–J) show neurons coexpressing PP2A‐29B dsRNA. The scale bar in (A) is 50 μm. (A, F) C4da neurons (co‐)expressing lacZ. (B, G) C4da neurons (co‐)overexpressing Sox14. (C, H) C4da neurons (co‐)overexpressing Mical. (D, I) C4da neurons (co‐)expressing MicalΔredox. (E, J) C4da neurons (co‐)expressing MicalΔCH.KPenetrance of pruning defects in panels (A–J). *P < 0.05, ***P < 0.0005, Fisher's exact test. Numbers of neurons (animals) for each genotype are given in the figure, and the numbers of animals are given below in parentheses.LSeverity of pruning defects in panels (A–J) as assessed by number of primary and secondary dendrites attached to soma at 18 h APF. Data are mean ± s.d., *P < 0.05, ***P < 0.0005, Wilcoxon's test. Numbers of neurons (animals) for each genotype are given in the figure.

During c4da neuron dendrite pruning, the steroid hormone ecdysone induces the expression of important pruning genes, in particular the transcription factor Sox14 and the actin‐severing enzyme Mical. We therefore tested next whether Sox14 or Mical might interact with PP2A during dendrite pruning. Overexpression of *Sox14* or *Mical* alone did not cause dendrite pruning defects (Fig 2A–C). However, *Sox14* overexpression significantly reduced the severity of the dendrite pruning defects induced by loss of *PP2A‐29B*, as assessed by the reduced number of dendrites attached to the soma under these conditions (Fig 2F, G, K, and L). Mical overexpression caused a significant reduction in both penetrance and severity of the pruning defects caused by *PP2A‐29B* knockdown (Fig 2H, K, and L). In order to address whether actin‐related domains of Mical, such as the oxidoreductase domain and the calponin homology (CH) domain, a putative actin binding site 28, are required for this genetic interaction, we next tested whether the expression of Mical mutants lacking these domains could also suppress the dendrite pruning defects induced by *PP2A‐29B* knockdown. Overexpression of *MicalΔredox* alone in *c4da* neurons did not cause dendrite pruning defects (Fig 2D, K, and L) but failed to suppress the effects of PP2A‐29B dsRNA (Fig 2I, K, and L). Interestingly, overexpression of *MicalΔCH* in c4da neurons alone had a dominant effect and caused strong dendrite pruning defects (Fig 2E, K, and L), possibly reflecting a direct involvement of this domain in the catalytic cycle of Mical. Consistently, coexpression of *MicalΔCH* with *PP2A‐29B* dsRNA strongly enhanced the severity of the dendrite pruning defects (Fig 2J–L). While Mical overexpression strongly suppressed the pruning defects caused by loss of PP2A, it did not significantly affect the larval dendrite defects (Fig EV1), suggesting that the mechanistic basis underlying these defects may not be identical. Taken together, these data indicate that the role of PP2A during dendrite pruning is linked to the ecdysone targets *Sox14* and *Mical*, and the actin‐modifying domains of Mical are crucial for this.

### Effects of PP2A on Sox14 and Mical expression

**Figure 3:**
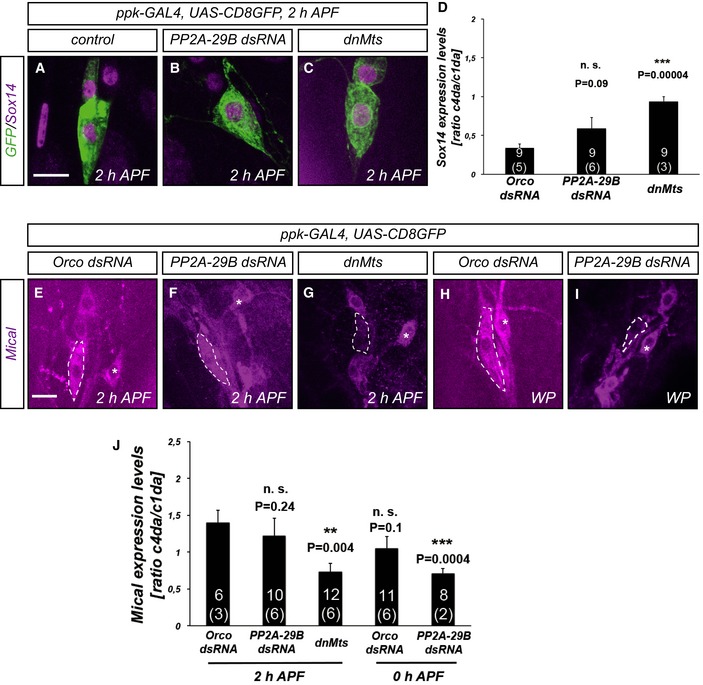
Effects of PP2A on ecdysone target gene expression during dendrite pruning C4da neurons were labeled by CD8::GFP expression under *ppk‐GAL4*, and the expression of Sox14 or Mical was assessed by immunofluorescence.
A–CSox14 stainings at 2 h APF. (A) Control c4da neurons. (B) C4da neurons expressing PP2A‐29B dsRNA. (C) C4da neuron expressing dominant‐negative Mts.DQuantification of Sox14 expression in (A–C). C4da neuron Sox14 expression was normalized to Sox14 expression in neighboring ddaE c1da neurons (identified by characteristic cell body shape). Data are mean ± s.d. The numbers of neurons (animals) for each genotype are given in each graph (data were not derived from independent experiments). n.s., not significant, ***P < 0.0005, Wilcoxon's test.E–IMical stainings at the indicated time points. (E) Mical expression in control c4da neurons at 2 h APF. (F) Mical expression in c4da neurons expressing dominant‐negative Mts at 2 h APF. (G) Mical expression in c4da neurons expressing PP2A‐29B dsRNA at 2 h APF. (H) Mical expression in c4da neurons expressing Orco dsRNA at the white pupal (WP) stage (0 h APF). (I) Mical expression in c4da neurons expressing PP2A‐29B dsRNA at the white pupal stage (0 h APF).JQuantification of Mical expression in (E–I). C4da neuron Mical expression was normalized to Mical expression in neighboring ddaE c1da neurons (marked by asterisks). Data are mean ± s.d. The numbers of neurons (animals) for each genotype are given in each graph (data were not derived from independent experiments). n.s., not significant, **P < 0.005, ***P < 0.0005, Wilcoxon's test.Data information: Scale bars in (A and E) are 10 μm.

Sox14 and Mical are both transcriptionally induced by the ecdysone receptor at the onset of the pupal stage 5. The observed genetic interactions between PP2A and these two factors could therefore reflect a defect in Sox14 and/or Mical expression (and hence a defect in ecdysone‐mediated gene expression). To address this possibility, we next assessed the effects of PP2A knockdown on Sox14 and Mical expression by immunofluorescence. At 2 h APF, Sox14 can be detected in the nucleus of control c4da neurons (Fig 3A). In order to downregulate PP2A, we next expressed either PP2A‐29B dsRNA or dominant‐negative Mts in c4da neurons and assessed the effects on Sox14 expression. While *PP2A‐29B* knockdown did not affect Sox14 expression (Fig 3B and D), Sox14 levels seemed to be increased upon dnMts expression (Fig 3C and D).

At 2 h APF, Mical is also robustly expressed in c4da neurons and localizes to the cytoplasm (Fig 3E). Mical was also normally expressed in neurons expressing PP2A‐29B dsRNA (Fig 3F and J). Surprisingly, many c4da neurons expressing dnMts showed strongly reduced Mical staining at this stage (Fig 3G and J). To address these potentially contradictory results, we reassessed Mical expression at the white pupal stage (0 h APF). At this stage, c4da neurons expressing PP2A‐29B dsRNA also displayed reduced Mical levels (Fig 3H–J). We interpret these data as indicating a 2‐h delay in Mical expression upon PP2A downregulation. However, dendrite severing and pruning take place between 5 h APF and 10 h APF. We assess pruning phenotypes at 18 h APF, and the pruning defects induced by *PP2A‐29B* knockdown are still highly penetrant and severe at this much later stage. We therefore conclude that the observed genetic interactions between PP2A and Sox14/Mical cannot be explained through a role of PP2A in Mical gene expression alone.

### PP2A‐linked pruning defects can be suppressed by cofilin overexpression

**Figure EV5:**
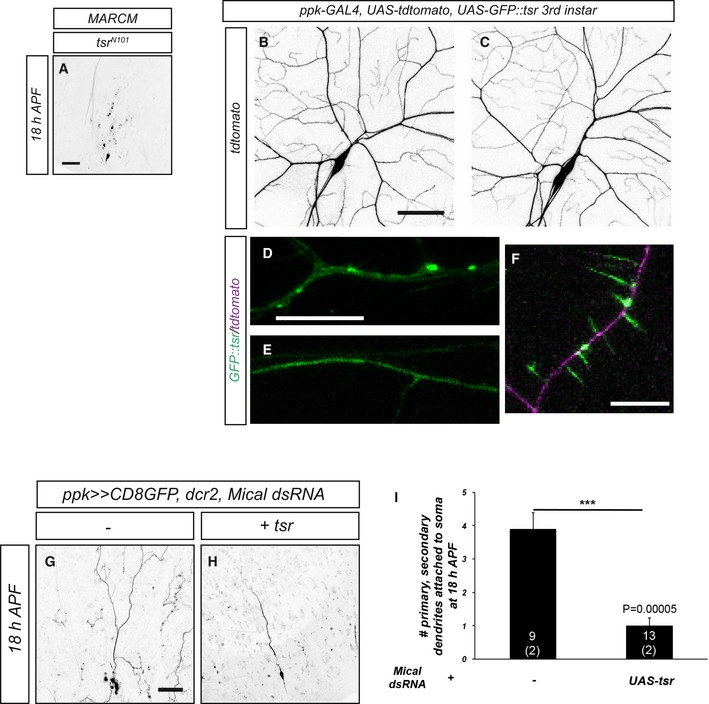
Characterization of GFP::tsr and Mical dsRNA AMorphology of c4da neuron MARCM clone homozygous for the cofilin/twinstar allele *tsr* ^*N121*^ at 18 h APF. 10/12 c4da neuron MARCM clones (from 9 animals) did not have dendrites attached to the soma anymore. Scale bar is 50 μm.B, CThe morphology of c4da neurons expressing GFP::tsr was visualized by tdTomato expression under *ppk‐GAL4*.D, EGFP::tsr distribution in a third‐instar larval dendrite of a control c4da neuron expressing Orco dsRNA (D) or in a dendrite of a c4da neuron expressing PP2A‐29B dsRNA (E). Only GFP signal is shown.FGFP::tsr signal in a third‐instar larval dendrite of a c3da neuron. Note the strong GFP signal in the dendritic spikes.G, HEffect of Mical knockdown on c4da neuron dendrite pruning at 18 h APF and genetic interaction with cofilin. (G) C4da neuron expressing Mical dsRNA. (H) C4da neuron coexpressing Mical dsRNA with UAS‐tsr.IQuantification of pruning defect severity in (G, H). Data are mean ± s.d., and n in the graph is the number of individual neurons (animals) assayed in the experiment. ***P < 0.0005, Wilcoxon's test.Data information: Scale bars are 50 μm in (A, B, and G), and scale bars are 10 μm in (D and F).

**Figure 4:**
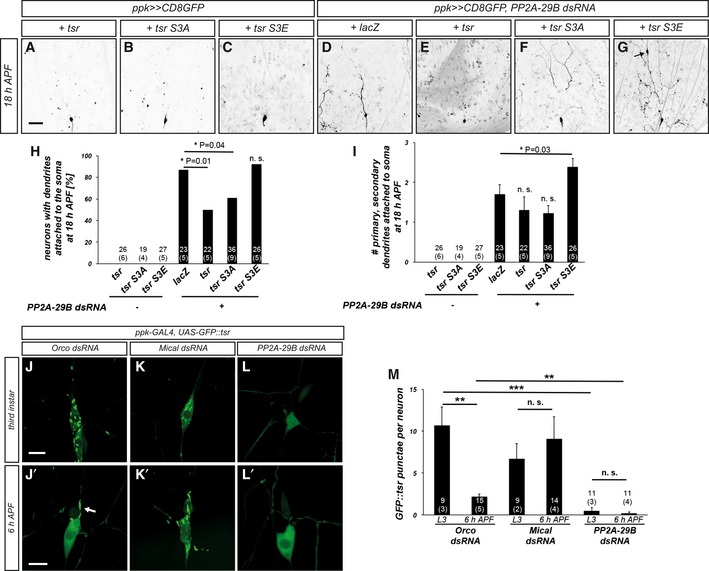
Interactions between PP2A and the actin regulator cofilin during c4da neuron dendrite pruning A–GThe indicated UAS‐tsr constructs were tested for their ability to suppress pruning defects induced by PP2A‐29B dsRNA expression. Suppression tests were performed as in Fig 2. Panels (A–C) show effects of the expression of wild‐type or phosphomutant tsr variants on dendrite pruning, and panels (D–G) show neurons coexpressing PP2A‐29B dsRNA. (A, E) C4da neuron (co‐)overexpressing wild‐type tsr. (B, F) C4da neuron (co‐)overexpressing tsr S3A. (C, G) C4da neuron (co‐)overexpressing tsr S3E. The arrow in panel G denotes a characteristic varicosity at the tip of an unpruned dendrite. (D) C4da neuron coexpressing PP2A‐29B dsRNA and lacZ as a UAS titration control.HPenetrance of pruning defects in panels (A–G). *P < 0.05, Fisher's exact test. Numbers of neurons (animals) for each genotype are given in the figure.ISeverity of pruning defects in panels (A–G) as assessed by number of primary and secondary dendrites attached to soma at 18 h APF. Data are mean ± s.d., *P < 0.05, Wilcoxon's test. Numbers of neurons (animals) for each genotype are given in the figure.J–LGFP‐tagged *Drosophila* cofilin (GFP::tsr) was expressed in c4da neurons under *ppk‐GAL4*, and its localization was assessed at the indicated developmental stages by live imaging. Panels (J–L) show GFP::tsr localization in c4da neurons at the third‐instar stage, and panels (J’–L’) show GFP::tsr localization in c4da neurons at 6 h APF. (J, J’) Control c4da neurons expressing Orco dsRNA. (K, K’) C4da neurons expressing Mical dsRNA. (L, L’) C4da neurons expressing PP2A‐29B dsRNA.MQuantification of GFP::tsr punctae in samples (J–L’). Data are mean ± s.d., and the numbers of neurons (animals) analyzed for each genotype are given in the figure (data were not derived from independent experiments). **P < 0.005, ***P < 0.0005, n.s., not significant, Wilcoxon's test.Data information: Scale bars are 50 μm in (A) and 10 μm in (J).

The observation that PP2A interacts with Mical during dendrite pruning prompted us to investigate whether PP2A could be linked to actin regulation during this process. We therefore asked whether upregulation of a different actin‐severing activity could also suppress the pruning defects caused by loss of PP2A. Cofilin is a well‐characterized actin depolymerization factor that can also functionally cooperate with Mical 29. We first asked whether *Drosophila* cofilin, encoded by *Twinstar* (*tsr*), was itself required for c4da neuron dendrite pruning. MARCM clones homozygous for the cofilin mutant allele *tsr* ^*N121*^ did not display dendrite pruning defects at 18 h APF (Fig EV5A). However, *tsr* overexpression caused a partial suppression of the pruning defects upon *PP2A‐29B* knockdown compared to the *lacZ* titration control (Fig 4A, D, E, H, and I).

Cofilin is inhibited by phosphorylation by LIM kinase 30, 31 and activated through dephosphorylation by slingshot phosphatases 32, and this is important for neuronal morphogenesis 33. Consequently, nonphosphorylatable versions of tsr are constitutively active, and phosphomimetic tsr mutant versions are inactive. Overexpression of UAS transgenes encoding nonphosphorylatable *tsr* ^*S3A*^ or phosphomimetic *tsr* ^*S3E*^ alone did not cause dendrite pruning defects (Fig 4B and C). UAS‐*tsr* ^*S3A*^ afforded a similar, but not better, suppression of the pruning defects induced by PP2A knockdown as UAS‐*tsr wt* (Fig 4F, H, and I), indicating that the phosphorylation state of cofilin does not affect its ability to suppress PP2A. UAS‐*tsr* ^*S3E*^ enhanced the severity of the pruning defects upon PP2A knockdown, consistent with a mild dominant or synergistic effect (Fig 4G–I).

We also wished to assess the localization of cofilin in c4da neurons. To this end, we used transgenic N‐terminally GFP‐tagged *Drosophila* tsr driven by the GAL4/UAS system (UAS‐GFP::tsr). GFP::tsr localized to multiple punctate, sometimes elongated structures in the soma and dendrites of c4da neurons during the larval stage (Figs 4J and M, and EV5). These GFP::tsr punctae were reminiscent of cofilin/actin aggregates that can be induced by cofilin overexpression or stress in mammalian cells 34. In support of the idea that these punctae contained actin, GFP::tsr also strongly localized to the dendritic “spikes” on the dendrites of c3da neurons that are known to be actin‐rich 35 (Fig EV5D) (the *ppk‐GAL4* insertion used for GFP::tsr expression in these experiments is also stochastically expressed in c3da neurons). At 6 h APF, most GFP::tsr punctae in c4da neurons had vanished and GFP::tsr now displayed a largely dispersed distribution (Fig 4J’ and M). Notably, one or two bright elongated GFP::tsr accumulations could still be seen at this stage that localized proximally to dendritic thinnings in primary dendrites (Fig 4J’). This localization was somewhat reminiscent of the localization of active cofilin in filopodia 36. C4da neurons expressing Mical dsRNA had a similar number of GFP::tsr punctae as control neurons at the larval stage (Fig 4K and M). However, several c4da neurons expressing Mical dsRNA still displayed many GFP::tsr accumulations at 6 h APF (Fig 4K’ and M), indicating that Mical was required for the observed changes in GFP::tsr localization between larvae and pupae. In contrast to control neurons, GFP::tsr was evenly dispersed in larval c4da neurons expressing PP2A‐29B dsRNA, with no punctae visible (Fig 4L and M). At 6 h APF, such GFP::tsr accumulations were also not seen at the bases of proximal dendrites in these neurons (Fig 4L’ and M). Thus, PP2A interacts genetically with actin disassembly factors and affects the localization of cofilin in c4da neurons.

### Loss of PP2A can be ameliorated by manipulation of actin regulators

**Figure 5:**
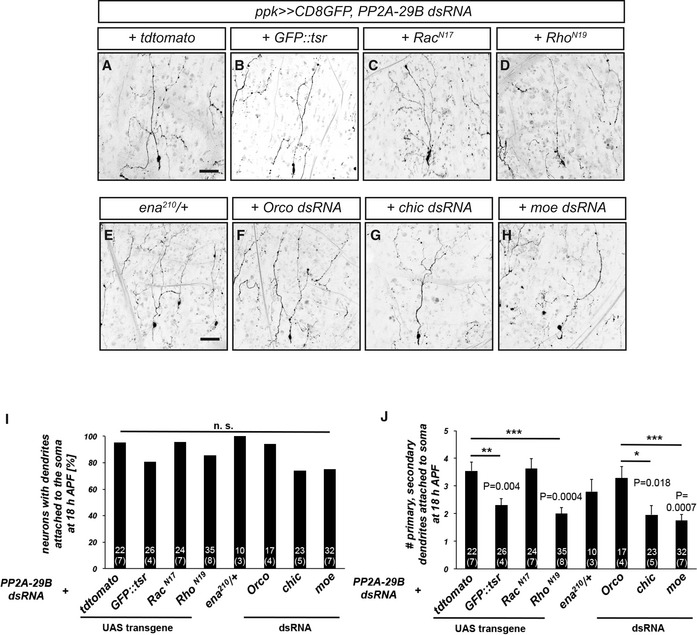
PP2A interacts with actin regulators during dendrite pruning A–HGenetic interactions between PP2A‐29B and actin regulators during dendrite pruning. The indicated manipulations of actin regulators were tested for their ability to suppress pruning defects induced by PP2A‐29B dsRNA expression. Suppression tests were performed as in Fig 2. (A) C4da neuron coexpressing PP2A‐29B dsRNA and tdTomato as suppression control. (B) C4da neuron coexpressing PP2A‐29B dsRNA and GFP::tsr. (C) C4da neuron coexpressing PP2A‐29B dsRNA and Rac^N17^. (D) C4da neuron coexpressing PP2A‐29B dsRNA and Rho^N19^. (E) C4da neuron expressing PP2A‐29B dsRNA in *ena* ^*210*^ */*+ background. (F) C4da neuron coexpressing PP2A‐29B dsRNA and Orco dsRNA as suppression control. (G) C4da neuron coexpressing PP2A‐29B dsRNA and Chicadee dsRNA. (H) C4da neuron coexpressing PP2A‐29B dsRNA and moesin dsRNA.IPenetrance of pruning defects in panels (A–H). n.s., not significant, Fisher's exact test. Numbers of neurons (animals) for each genotype are given in the figure.JSeverity of pruning defects in panels (A–H) as assessed by number of primary and secondary dendrites attached to soma at 18 h APF. Data are mean ± s.d., *P < 0.05, **P < 0.005, ***P < 0.0005, Wilcoxon's test. Numbers of neurons (animals) for each genotype are given in the figure.Data information: Scale bars in (A and E) are 50 μm.

In order to deepen the links between PP2A and actin regulation during dendrite pruning, we next sought to identify additional genetic interactions between PP2A and actin. To this end, we downregulated known actin regulators and asked whether this could ameliorate the pruning defects caused by loss of PP2A. As a control, we expressed GFP::tsr in this background. While GFP::tsr did not reduce the penetrance of the pruning defects induced by PP2A‐29 dsRNA, when compared to overexpression of tdTomato, it decreased their severity (Fig 5A, B, I, and J). This suppression was lower than with untagged tsr, but this could be due to lower expression levels of GFP::tsr (this construct is a site‐directed transgene which in our experience has lower expression when compared to randomly inserted ones). Rac family GTPases are another prominent class of actin regulators. While inhibition of Rac1 with a dominant‐negative construct did not ameliorate the dendrite pruning defects induced by PP2A‐29 dsRNA, inhibition of Rho with a dominant‐negative construct reduced the severity of the pruning defects significantly, such that each neuron had fewer dendrite branches attached to the soma at 18 h APF (Fig 5C, D, I, and J). In order to manipulate additional regulators of actin polymerization, we next used mutants or dsRNA constructs. Partial loss of the actin polymerase *enabled* in a heterozygous *ena* ^*210*^/+ mutant background did not have an effect on the severity of the pruning defects (Fig 5E, I, and J). Downregulation of profilin, an actin binding protein involved in the regulation of actin dynamics, significantly reduced the effects of PP2A‐29B dsRNA when compared to an Orco control dsRNA (Fig 5F, G, I, and J). The strongest suppression of the severity of the pruning defects was achieved by downregulation of the cortical actin regulator moesin (Fig 5H–J).

**Figure EV6:**
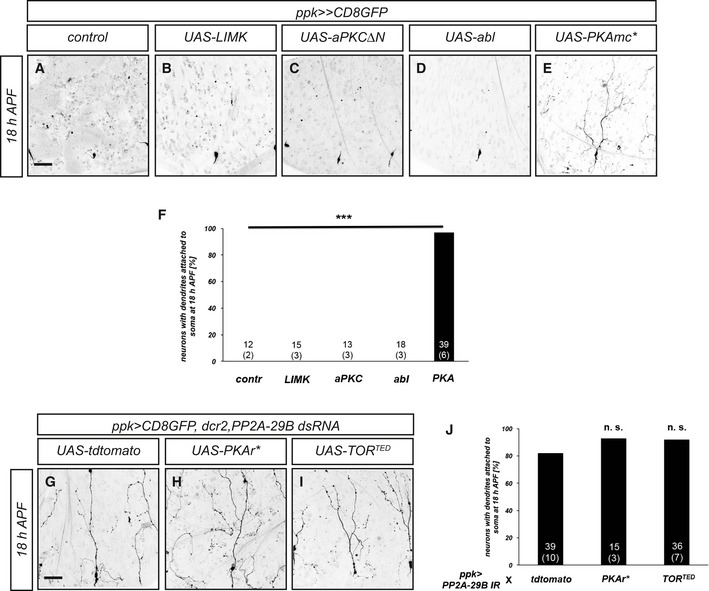
Kinases as potential negative pruning regulators A–EThe indicated kinase constructs were expressed in c4da neurons, and effects on dendrite pruning were assessed at 18 h APF. (A) Control c4da neuron. (B) C4da neuron expressing LIM kinase. (C) C4da neuron expressing activated atypical kinase C (aPKCΔN). (D) C4da neuron expressing abl. (E) C4da neuron expressing the active PKA catalytic subunit (PKAmc*).FPenetrance of pruning defects in (A–E). Numbers of neurons (animals) for each genotype are given in the graph. ***P < 0.0005, Fisher's exact test.G–IInhibition of PKA or TOR does not suppress the pruning defects induced by PP2A‐29B dsRNA expression. C4da neurons were labeled by CD8GFP expression under *ppk‐GAL4*, and dendrite pruning defects were assessed at 18 h APF. (G) C4da neuron coexpressing PP2A‐29B dsRNA and tdTomato as a titration control. (H) C4da neuron coexpressing PP2A‐29B dsRNA and PKAr* (constitutively active PKA regulatory subunit). (I) C4da neuron coexpressing PP2A‐29B dsRNA and dominant‐negative TOR^TED^.JPenetrance of pruning defects in (G–I). Numbers of neurons (animals) for each genotype are given in the graph. n.s. not significant, Fisher's exact test.Data information: Scale bars in (A and G) are 50 μm.

We also performed an overexpression survey to identify possible kinases counteracting PP2A. While overexpression of LIM kinase, active atypical protein kinase C (aPKC), or abelson kinase (abl, a tyrosine kinase), did not cause dendrite pruning defects, overexpression of a constitutively active form of protein kinase A (PKA mc*) caused strong and fully penetrant c4da neuron dendrite pruning defects (Fig EV6). However, neither inhibition of PKA nor the previously identified negative pruning regulator TOR 37 could suppress the pruning defects caused by PP2A knockdown (Fig EV6).

Taken together, our results suggest that PP2A regulates one or several aspects of actin dynamics and/or cortical actin organization that are required for dendrite pruning.

## Discussion

**Figure 6:**
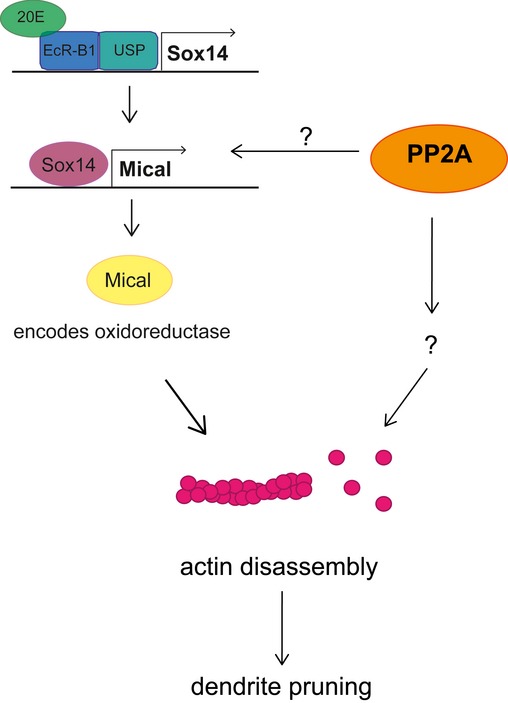
Hypothetical model for PP2A action during dendrite pruning Our data indicate a two‐pronged effect of PP2A on pruning: firstly, PP2A is required for timely expression of Mical via an unknown mechanism. Secondly, PP2A appears to affect actin dynamics in neurons during both the larval and pupal stages.

In this study, we identified an important role for the protein phosphatase PP2A during large‐scale dendrite pruning of *Drosophila* c4da neurons during metamorphosis. Our data indicate that a PP2A holoenzyme containing either the Wdb or Tws regulatory subunit is involved in pruning regulation. Our data strongly link PP2A to actin regulation during dendrite pruning. In particular, our genetic interaction studies show that the pruning defects caused by loss of PP2A can be suppressed by overexpression of the actin‐severing enzyme Mical. Mical is induced by ecdysone at the onset of the pupal stage and is required for c4da neuron dendrite pruning. While our data and an accompanying study by Yu and colleagues 38 show that induction of Mical expression is delayed by loss of PP2A, this effect is transient, and there is no strong effect on Mical expression at 2 h APF, when other mutants that directly affect Mical expression still show strongly reduced Mical levels (e.g., Ref. 39, 40). While the delay in Mical expression could contribute to the observed pruning defects, several lines of evidence indicate that PP2A has an additional function in actin regulation, such as the observations that the actin‐related domains of Mical are required for suppression of the PP2A phenotype (Fig 2) and that overexpression of cofilin can also partially rescue the PP2A phenotype (Fig 4). Furthermore, our genetic analyses (Fig 5) link PP2A to actin dynamics and cortical actin organization. In this context, it is interesting that c4da neurons lacking PP2A had an altered distribution of GFP::tsr (GFP‐tagged cofilin) (Fig 4). This transgene localized to punctate structures upon GAL4‐mediated expression. It is known that active cofilin can co‐assemble with actin into so‐called cofilin rods that resemble actin/cofilin structures at the base of filopodia where actin turnover is high 36. As GFP::tsr also localizes to the actin‐rich dendritic spikes of c3da neurons (Fig EV5), it is interesting to speculate that the formation of these structures may in part reflect the overall F‐actin amount in the cell, or a cell's propensity to form F‐actin. The lack of GFP::tsr punctae in c4da neurons lacking PP2A might therefore indicate globally altered actin dynamics (Fig 4). These results will be important leads in order to better define the role of PP2A and actin regulation during dendrite pruning in the future. Since the data presented by us and others 23, 24 suggest that loss of PP2A affects actin dynamics and hence possibly both F‐actin assembly and disassembly, it is interesting to speculate that the underlying causes for the larval dendrite morphogenesis defects and the pruning defects may be similar. We therefore tentatively place PP2A in a pathway at least partially in parallel to Mical during dendrite pruning (Fig 6).

While our genetic data suggest that PP2A is not involved in the temporal regulation of microtubule disassembly via the Par‐1/tau pathway (Fig EV3), the parallel study by the Yu lab 38 found that loss of PP2A leads to a defect in dendritic microtubule orientation. We have previously found that the uniform plus end‐in orientation of dendritic microtubules is required for dendrite pruning 14. It is therefore conceivable that these microtubule‐based defects also contribute to the observed dendrite pruning defects. Which of the observed defects in gene expression, microtubule orientation and actin dynamics contributes most to the observed defects remains to be seen. However, the combination of the two studies clearly establishes PP2A as a broad and important regulator of the neuronal cytoskeleton during development.

## Materials and Methods

### Fly strains

All crosses were done at 25°C under standard conditions. For expression in c4da neurons, we used *ppk‐GAL4* insertions on the second and third chromosomes 41. MARCM clones were induced with *SOP‐FLP* 42 and labeled by tdTomato expression under *nsyb‐GAL4* ^*R57C10*^ 43. Here, the PP2A mutant allele PP2A‐29B^GE16781^ 26 was recombined with *FRT40A*. Other fly lines were UAS‐Mical 44, UAS‐MicalΔCH, UAS‐MicalΔRedox 27, UAS‐Sox14 39, UAS‐tdtomato 17, UAS‐tsr, UAS‐tsrS3A, UAS‐tsrS3E (BL #9235, 9237, 9239) 32, UAS‐lacZ, UAS‐lifeAct::GFP (BL 35544), UAS‐dnMts 45, UAS‐PAR‐1 9, UAS‐Venus‐kat60L1 46, tau^KO^ 47, *FRT G13, tsr* ^*N121*^ (BL #9109), *FRT 82B, wdb* ^*14*^ (BL #53712) 45, UAS‐aPKCΔN (BL #51673) UAS‐RacN17 (BL #6292), UAS‐RhoN19 (BL #7328), and ena^210^/CyO (BL #25404). UAS‐dsRNA lines were as follows: PP2A‐29B (BL #29384), Wrd (BL #30512), Tws (VDRC# 34340), chicadee (VDRC #102759), moesin (VDRC #37917). Orco dsRNA (BL #31278) was used as control. UAS‐dsRNA lines were used with UAS‐dicer2 48. For conditional Tws and CG4733 CRISPR 49, we used UAS‐Cas9P2 (BL #58986). For kinase manipulations, we used UAS‐PKA mC* and UAS‐PKAr* 50, UAS‐LIMK 31, UAS‐abl 51, and UAS‐TOR^TED^ 52.

### Cloning and transgenes

^*HA*^ *PP2A‐29B* and *GFP::tsr* were cloned into pUAST attB by standard methods. Transgenes were injected in flies carrying the 86Fb acceptor site 53.

### Dissection, microscopy, and live imaging

For analysis of pruning defects, pupae were dissected out of the pupal case at 18 h APF and analyzed live using a Zeiss LSM 710 confocal microscope. For *PP2A‐29B* genetic interactions, candidate modifiers were crossed to a second chromosome insertion of *ppk‐GAL4* combined with UAS‐CD8GFP and UAS‐dicer2 and *UAS‐PP2A‐29B dsRNA* on the third chromosome (BL# 29384). UAS‐GFP::tsr was expressed under *ppk‐GAL4* and imaged live in ether‐anaesthetized third‐instar larvae or at 6 h APF. All images were analyzed using Fiji 54.

### Antibodies and immunohistochemistry

Pupal body wall filets were dissected quickly in PBS and fixed in PBS containing 4% formaldehyde for 20 minutes. Chicken anti‐GFP antibodies were from Aves labs, respectively. Other antibodies were guinea pig anti‐Sox14 55 and rabbit anti‐Mical 40.

### Quantification and statistical analysis

Pruning phenotypes in Figs 1, 2, 4, and 5 were analyzed by determining the fraction of neurons that still had dendrites attached to the soma to reflect the phenotypic penetrance, and these data were analyzed using a two‐tailed Fisher's exact test. To assess severity, we also counted the number of primary and secondary branches (i.e., the sum of all primary and secondary dendrites per neuron) still attached to the soma at 18 h APF. These data were analyzed using Wilcoxon's test. In a similar way, we determined the number of neurons with unusual varicosities in Fig 5A–C.

For quantification of immunofluorescence experiments, c4da neuron staining intensities were measured in Fiji and normalized to c1da neuron staining intensities in the same image. These data were analyzed using Wilcoxon's test.

The number of GFP::tsr punctae was mean ± s.d. and analyzed using Wilcoxon's test. For dendritic field coverage analysis, dendritic fields were defined by segment border denticles (in the anterior/posterior direction), the dorsal midline, and the middle between dorsal and lateroventral c4da neuron dendrites (in the dorsoventral direction). Actual coverage was calculated by connecting outer dendrite terminals with each other as in Ref. 35. The covered area was then compared to the maximal dendritic field.

## Author contributions

NW and SR designed and conceived the project and interpreted the data. NW and SR performed experiments. NW, UG, and SR contributed reagents. SR wrote the manuscript.

## Conflict of interest

The authors declare that they have no conflict of interest.

## Supporting information



Expanded View Figures PDF

Review Process File
