# Supplementary material for: PP2A phosphatase is required for dendrite pruning via actin regulation in Drosophila
Source: EMBO Rep. 2020 Mar 24;21(5):e48870. doi: 10.15252/embr.201948870 (PMC7202059; doi:10.15252/embr.201948870)
Supplement: Supplementary file 1 — Expanded View Figures PDF [file EMBR-21-e48870-s001.pdf]

Expanded View Figures

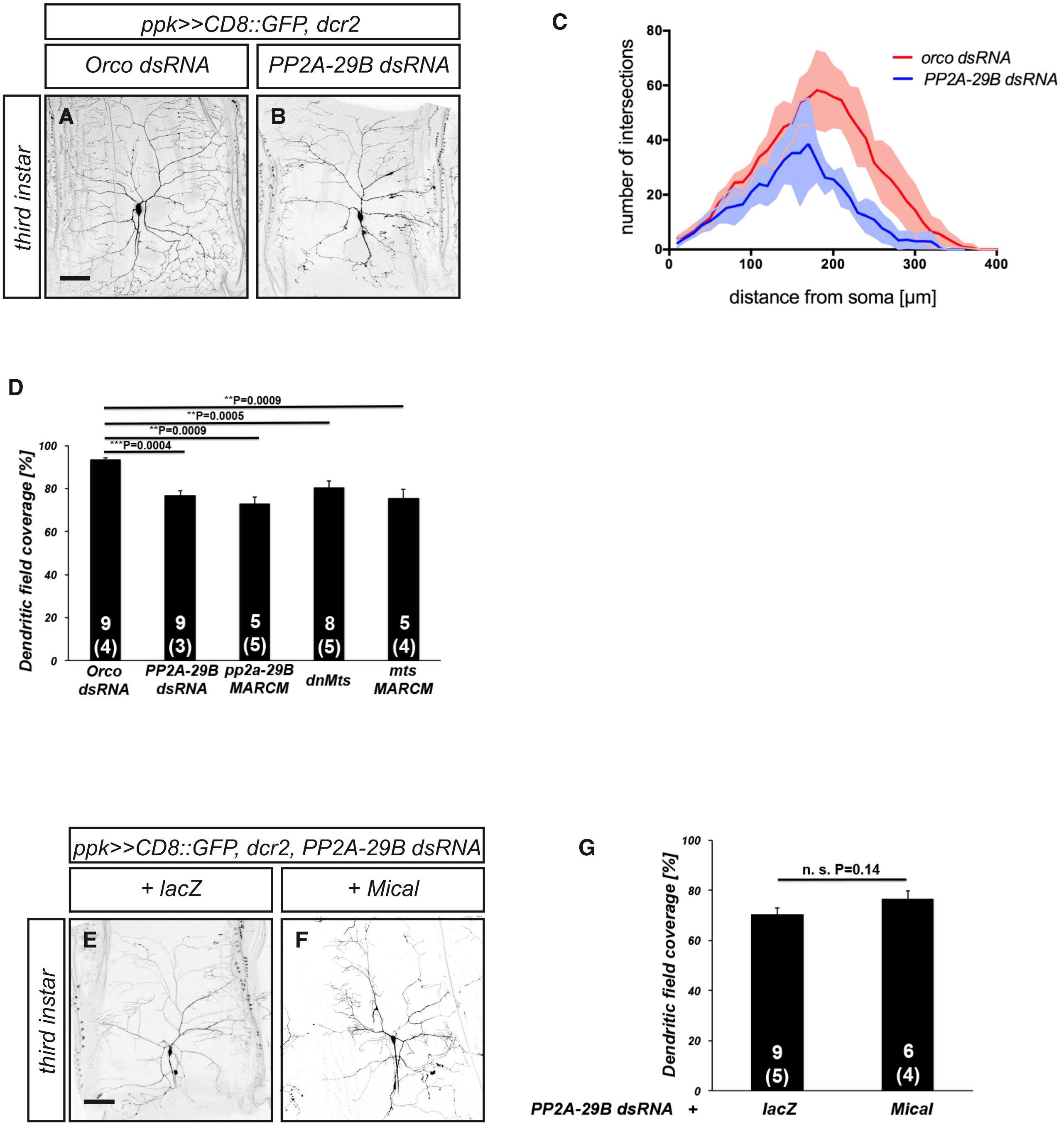

Figure EV1.

Figure EV1. Effects of PP2A-29B knockdown on larval dendrite morphology.

A, B Morphology of control c4da neurons (A) or c4da neurons expressing PP2A-29B dsRNA under *ppk-GAL4* (B) at the third-instar larval stage. Scale bar is 100 μm.

C Sholl analysis of dendrite branch number and distribution for control c4da neurons and c4da neurons expressing PP2A-29B dsRNA. N was 9 neurons (from 3 animals) for Orco dsRNA and 5 neurons (from 2 animals) for PP2A-29B dsRNA, respectively.

D Dendritic field coverage of control c4da neurons and c4da neurons upon PP2A manipulation, respectively. Neurons for genotypes other than the Orco dsRNA control and PP2A-29B dsRNA include those shown in Fig 1 C, E, F. The number of neurons (animals) in each sample is given in the graph. Data are mean ± s.d., and the *P* value was calculated using Wilcoxon's test.

E, F Morphology of c4da neurons coexpressing PP2A-29B and lacZ (E) or c4da neurons coexpressing PP2A-29B and Mical (F) at the third-instar larval stage. Scale bar is 100 μm.

G Dendritic field coverage of c4da neurons in (E and F). The number of neurons (animals) in each sample is given in the graph. Data are mean ± s.d.. n.s., not significant, Wilcoxon's test.

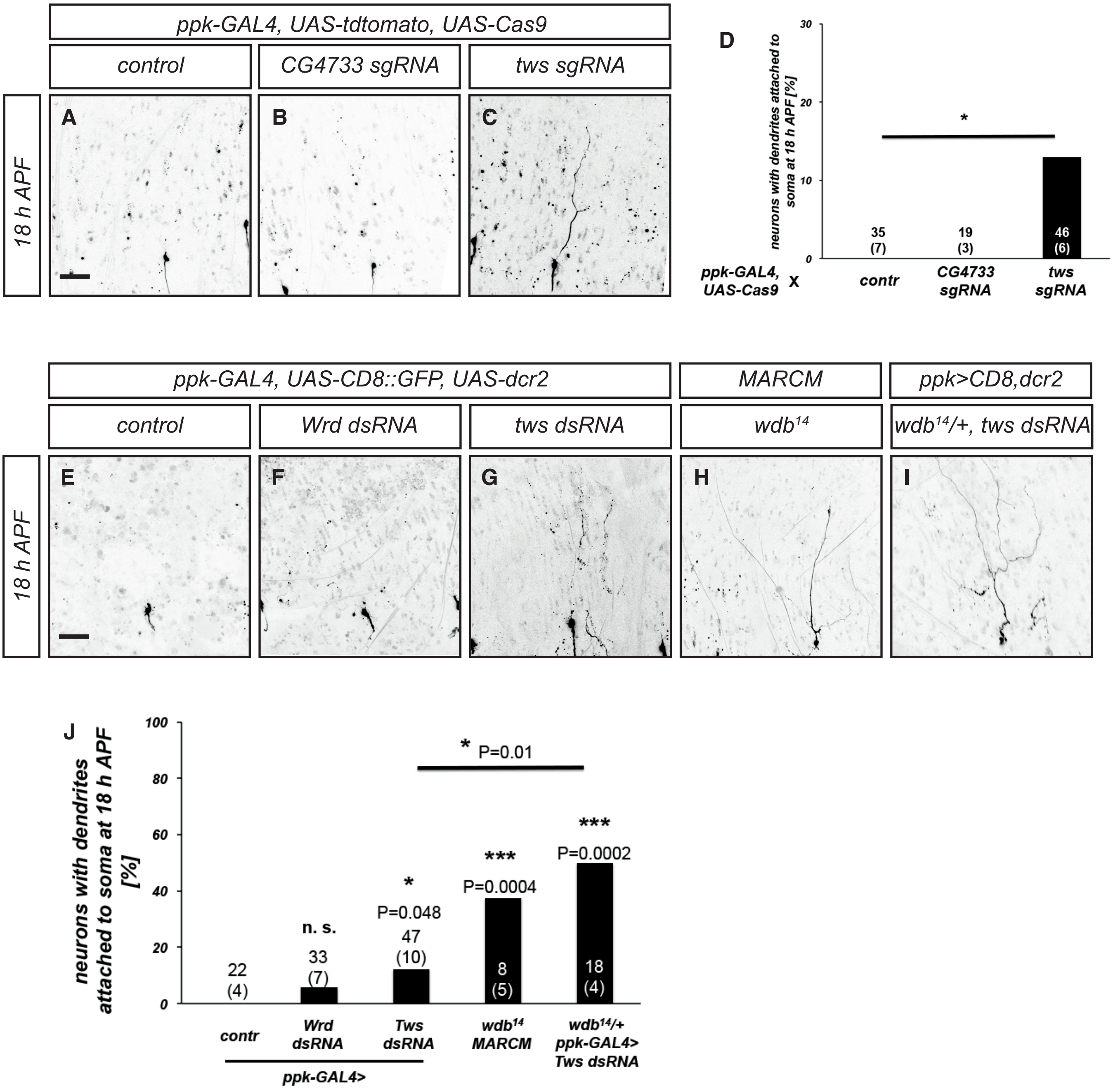

Figure EV2.

 © 2020 The Authors

Figure EV2. PP2A B subunits required for dendrite pruning.

A–C Morphology of c4da neurons expressing the indicated CRISPR/Cas9 constructs at 18 h after puparium formation (APF). C4da neurons were labeled by expression of tdTomato under *ppk-GAL4*. (A) Control c4da neuron expressing UAS-Cas9. (B) C4da neuron expressing UAS-Cas9 and an sgRNA construct against PR72/CG4733. (C) C4da neuron expressing UAS-Cas9 and an sgRNA construct against Twins/Tws.

D Penetrance of pruning defects at 18 h APF in (A–C). Numbers of neurons (animals) for each genotype are given in the figure. *$P < 0.05$, Fisher's exact test.

E–I Morphology of c4da neurons of the indicated genotypes at 18 h after puparium formation (APF). C4da neurons were labeled by the expression of CD8::GFP under *ppk-GAL4*, or by tdTomato expression in MARCM clones. (E) Control c4da neuron not expressing a dsRNA construct. (F) C4da neuron expressing wrd dsRNA under *ppk-GAL4*. (G) C4da neuron expressing tws dsRNA under *ppk-GAL4*. (H) C4da neuron MARCM clone homozygous for the wdb^{14} mutation. (I) C4da neuron expressing tws dsRNA under *ppk-GAL4* in a wdb^{14}/+ heterozygous mutant background.

J Penetrance of pruning defects at 18 h APF in (E–I). Numbers of neurons (animals) for each genotype are given in the figure. *$P < 0.05$, ***$P < 0.0005$, Fisher's exact test.

Data information: The scale bars in (A and E) are 50 μm.

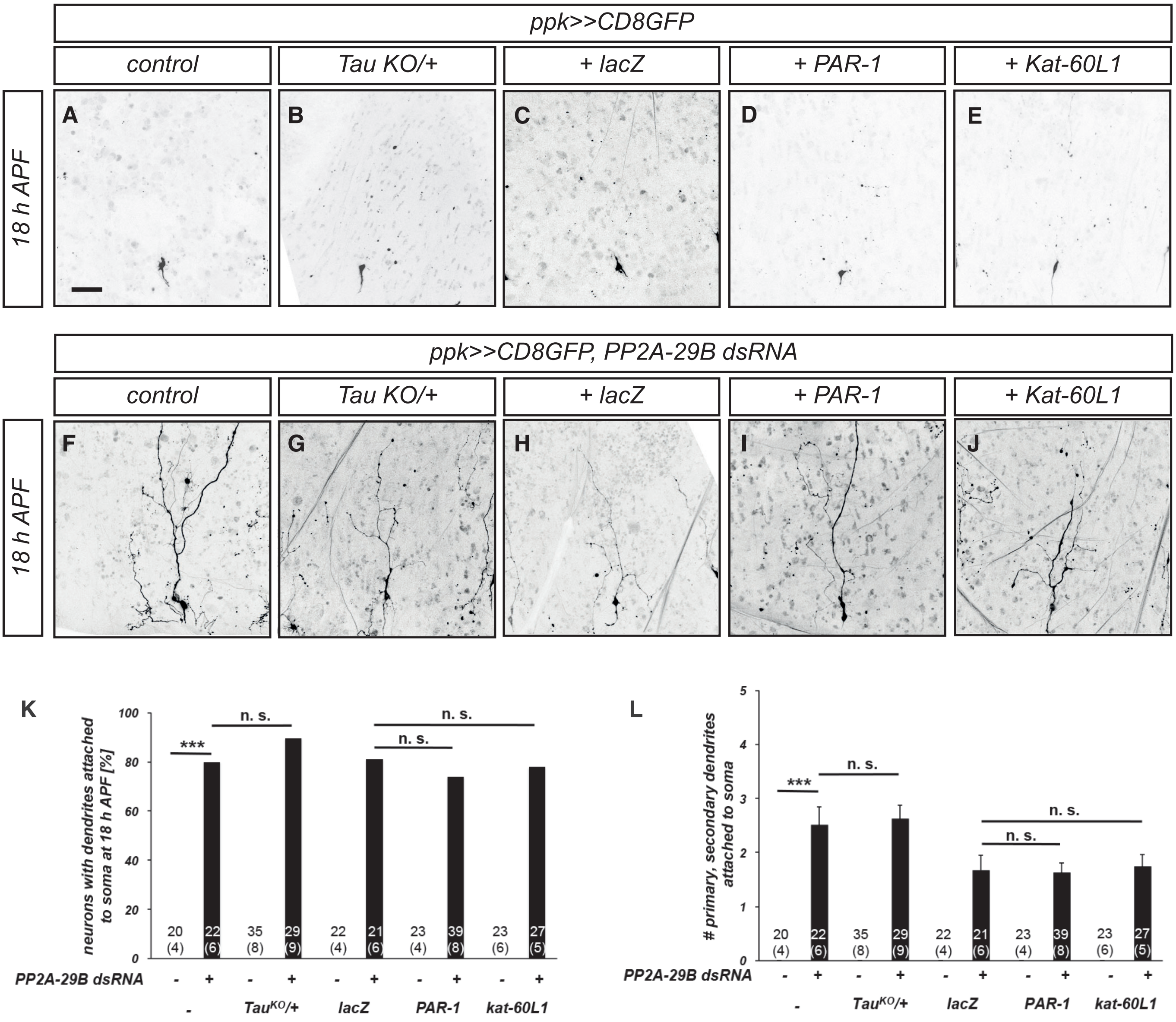

Figure EV3.

Figure EV3. The role of PP2A during c4da neuron dendrite pruning is not linked to Par-1-mediated microtubule disassembly.

A–J Microtubule pathway components were tested for their ability to suppress pruning defects induced by PP2A-29B dsRNA expression. C4da neurons were labeled by CD8GFP expression under *ppk-GAL4*, and dendrite pruning defects were assessed at 18 h APF. Panels (A–E) show effects of microtubule pathway manipulations alone, and panels (F–J) show neurons coexpressing PP2A-29B dsRNA. Scale bar in A is 50 μm. (A, F) Control c4da neurons. (B, G) C4da neurons in Tau^{KO}/+ heterozygous background. (C, H) C4da neurons (co-)expressing UAS-lacZ. (D, I) C4da neurons (co-)expressing PAR-1. (E, J) C4da neurons (co-)expressing ^{Venus}Kat-60L1.

K Penetrance of pruning defects in (A–J). ****P* < 0.0005, n.s., not significant, Fisher's exact test. Numbers of analyzed neurons (animals) are given in the graph.

L Severity of pruning defects in (A–J) at 18 h APF as assessed by number of primary and secondary dendrites attached to soma at 18 h APF. Data are mean ± s.d., and numbers of analyzed neurons (animals) are given in the graph. ****P* < 0.0005, Wilcoxon's test, n.s., not significant.

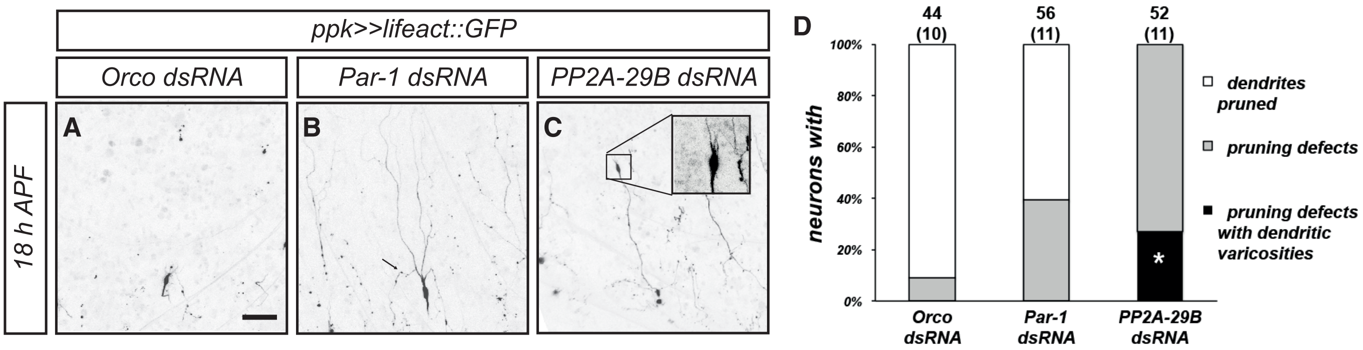

Figure EV4. Loss of PP2A causes unusual dendritic varicosities.

A–D Loss of PP2A induces large distal varicosities in unpruned dendrites at 18 h APF. The F-actin reporter lifeact::GFP was expressed under *ppk-GAL4* in c4da neurons expressing the indicated dsRNA constructs and imaged at 18 h APF. (A) Orco dsRNA control. (B) Par-1 dsRNA. The arrow denotes a small varicosity in a proximal dendrite. (C) PP2A-29B dsRNA. The inset shows an enlarged image of a large varicosity at the tip of an unpruned dendrite. (D) Quantification of pruning defects and dendritic varicosities in (A–C). Categories are as follows: neurons with pruned dendrites (white), neurons with dendrite pruning defects (i.e., still attached to soma) (gray), and neurons with pruning defects and big distal varicosities (black). For the last category, we compared Par-1 dsRNA with PP2A-29B dsRNA. The number of neurons (and number of animals) for each genotype is given in each graph (data were not derived from independent experiments). **P* < 0.05, Fisher's exact test.

 © 2020 The Authors

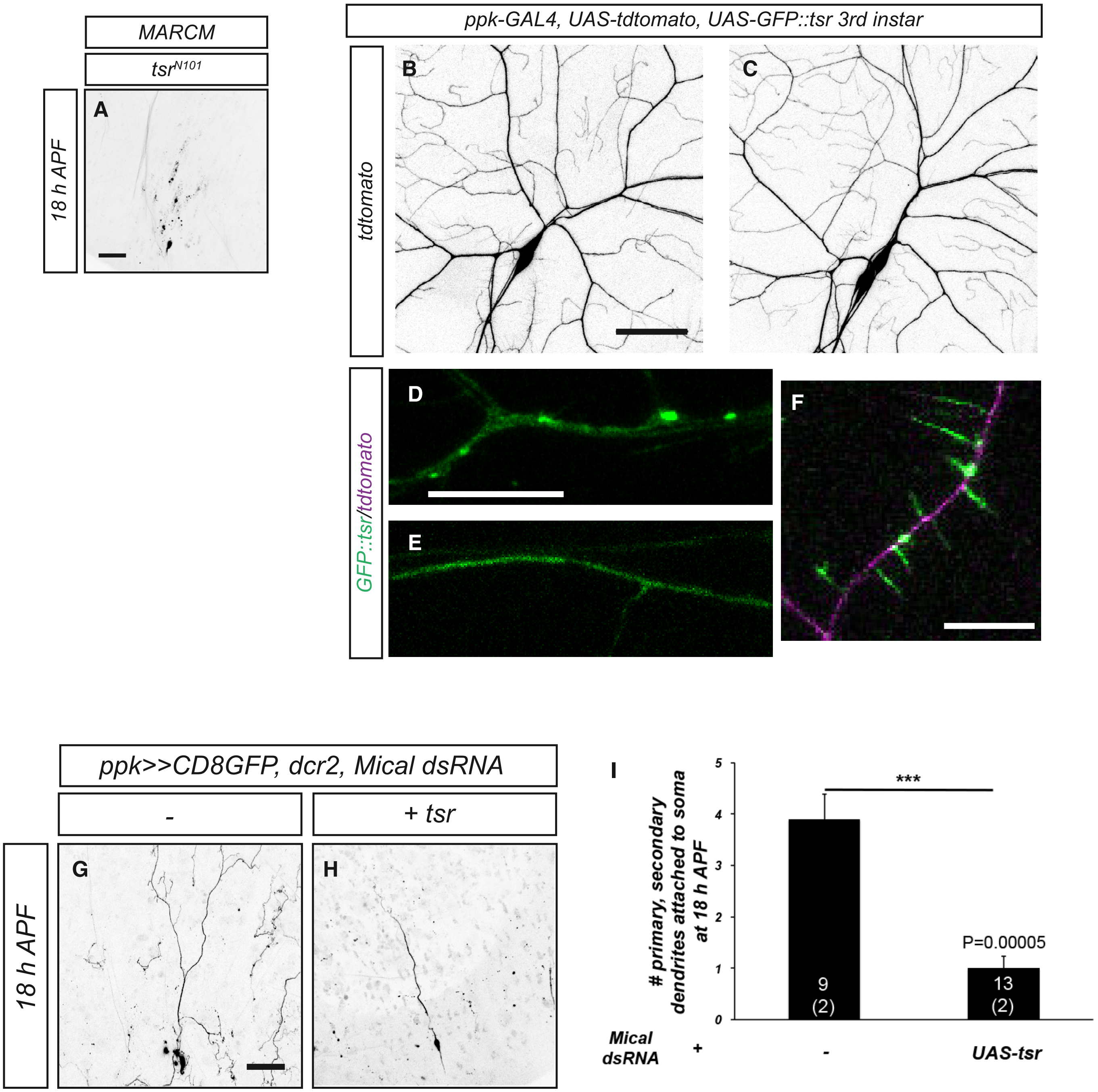

Figure EV5. Characterization of GFP::tsr and Mical dsRNA.

A Morphology of c4da neuron MARCM clone homozygous for the cofilin/twinstar allele *tsr*^{N121} at 18 h APF. 10/12 c4da neuron MARCM clones (from 9 animals) did not have dendrites attached to the soma anymore. Scale bar is 50 μm.

B, C The morphology of c4da neurons expressing GFP::tsr was visualized by tdTomato expression under *ppk-GAL4*.

D, E GFP::tsr distribution in a third-instar larval dendrite of a control c4da neuron expressing Orco dsRNA (D) or in a dendrite of a c4da neuron expressing PP2A-29B dsRNA (E). Only GFP signal is shown.

F GFP::tsr signal in a third-instar larval dendrite of a c3da neuron. Note the strong GFP signal in the dendritic spikes.

G, H Effect of Mical knockdown on c4da neuron dendrite pruning at 18 h APF and genetic interaction with cofilin. (G) C4da neuron expressing Mical dsRNA. (H) C4da neuron coexpressing Mical dsRNA with UAS-tsr.

I Quantification of pruning defect severity in (G, H). Data are mean ± s.d., and n in the graph is the number of individual neurons (animals) assayed in the experiment. ****P* < 0.0005, Wilcoxon's test.

Data information: Scale bars are 50 μm in (A, B, and G), and scale bars are 10 μm in (D and F).

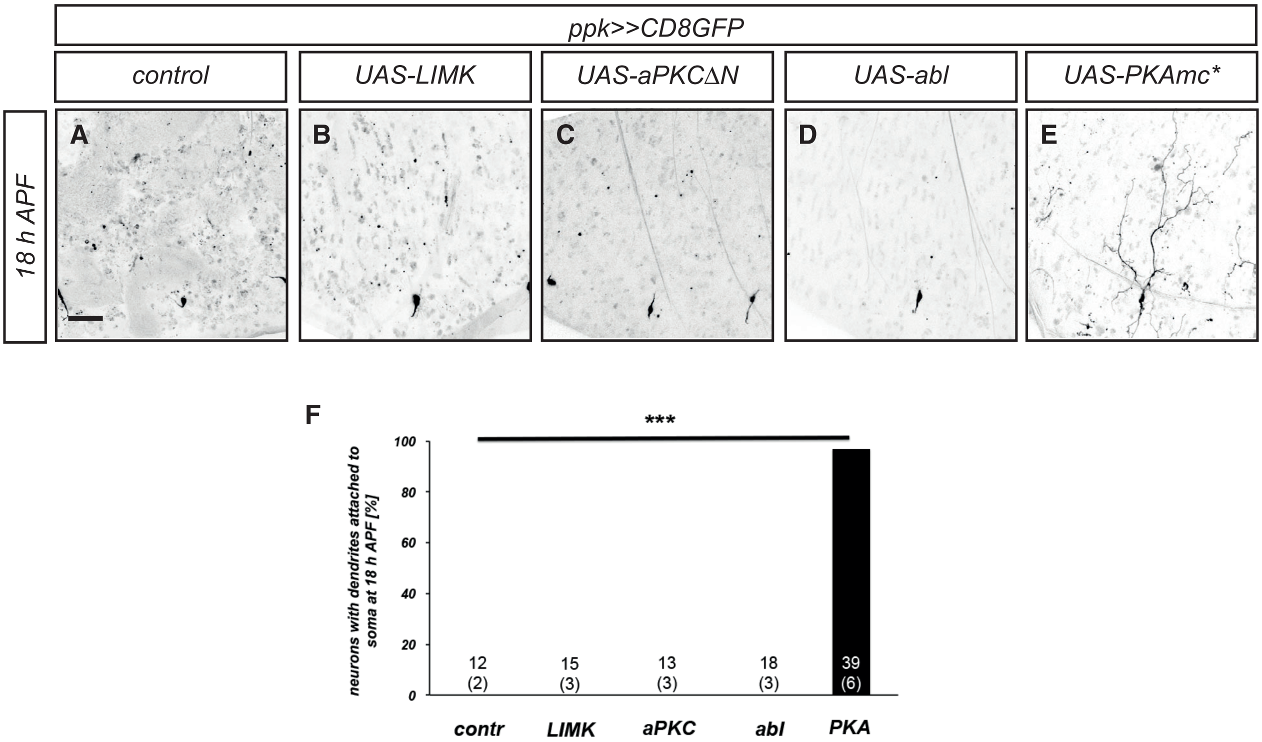

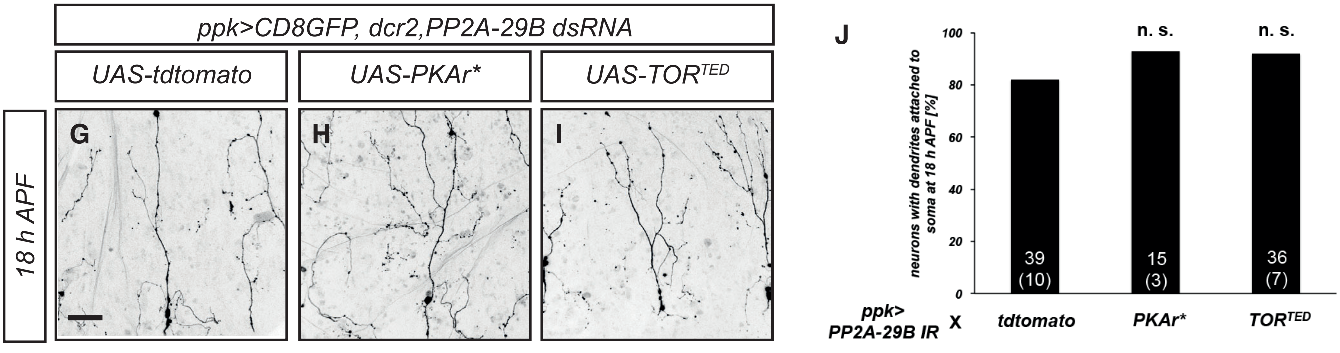

Figure EV6. Kinases as potential negative pruning regulators.

A–E The indicated kinase constructs were expressed in c4da neurons, and effects on dendrite pruning were assessed at 18 h APF. (A) Control c4da neuron. (B) C4da neuron expressing LIM kinase. (C) C4da neuron expressing activated atypical kinase C (aPKCΔN). (D) C4da neuron expressing abl. (E) C4da neuron expressing the active PKA catalytic subunit (PKAmc*).

F Penetrance of pruning defects in (A–E). Numbers of neurons (animals) for each genotype are given in the graph. ***$P < 0.0005$, Fisher's exact test.

G–I Inhibition of PKA or TOR does not suppress the pruning defects induced by PP2A-29B dsRNA expression. C4da neurons were labeled by CD8GFP expression under *ppk-GAL4*, and dendrite pruning defects were assessed at 18 h APF. (G) C4da neuron coexpressing PP2A-29B dsRNA and tdTomato as a titration control. (H) C4da neuron coexpressing PP2A-29B dsRNA and PKAr* (constitutively active PKA regulatory subunit). (I) C4da neuron coexpressing PP2A-29B dsRNA and dominant-negative TOR^{TED}.

J Penetrance of pruning defects in (G–I). Numbers of neurons (animals) for each genotype are given in the graph. n.s. not significant, Fisher's exact test.

Data information: Scale bars in (A and G) are 50 μm.

 © 2020 The Authors
