# Supplementary material for: PP2A phosphatase is required for dendrite pruning via actin regulation in Drosophila
Source: EMBO Rep. 2020 Mar 24;21(5):e48870. doi: 10.15252/embr.201948870 (PMC7202059; doi:10.15252/embr.201948870)
Supplement: Supplementary file 2 — Review Process File [file EMBR-21-e48870-s002.pdf]

EMBO *reports*

# PP2A Phosphatase Is Required for Dendrite Pruning via Actin Regulation in *Drosophila*

Neele Wolterhoff, Ulrike Gigengack, Sebastian Rumpf

**Review timeline:**

| | |
|---|---|
| Submission date: | 15 July2019 |
| Editorial Decision: | 4 September 2019 |
| Revision received: | 5 January 2020 |
| Editorial Decision: | 12 February 2020 |
| Revision received: | 27 February 2020 |
| Accepted: | 4 March 2020 |

Editor: Esther Schnapp

## Transaction Report:

(Note: With the exception of the correction of typographical or spelling errors that could be a source of ambiguity, letters and reports are not edited. The original formatting of letters and referee reports may not be reflected in this compilation.)

1st Editorial Decision 4 September 2019

Thank you for the submission of your manuscript to EMBO reports. I am sorry for the delay in getting back to you which is due to seasonal traveling. We have now received the full set of referee reports that is pasted below.

As you will see, all referees acknowledge that the findings are potentially interesting. However, they also all raise several technical concerns and point out that significant revisions are required and that the 2 back to back studies should be better aligned. I think that all concerns make sense and should therefore be addressed. Please let me know if you disagree and we can discuss the revisions further.

I would thus like to invite you to revise your manuscript with the understanding that the referee concerns must be fully addressed and their suggestions taken on board. Please address all referee concerns in a complete point-by-point response. Acceptance of the manuscript will depend on a positive outcome of a second round of review. It is EMBO reports policy to allow a single round of major revision only and acceptance or rejection of the manuscript will therefore depend on the completeness of your responses included in the next, final version of the manuscript.

Revised manuscripts should be submitted within three months of a request for revision; they will otherwise be treated as new submissions. Please contact us if a 3-months time frame is not sufficient for the revisions so that we can discuss this further. You can either publish the study as a short report or as a full article. For short reports, the revised manuscript should not exceed 27,000 characters (including spaces but excluding materials & methods and references) and 5 main plus 5 expanded view figures. The results and discussion sections must further be combined, which will help to shorten the manuscript text by eliminating some redundancy that is inevitable when discussing the same experiments twice. For a normal article there are no length limitations, but it should have more than 5 main figures and the results and discussion sections must be separate. In both cases, the entire materials and methods must be included in the main manuscript file.

Regarding data quantification, please specify the number "n" for how many independent experiments were performed, the bars and error bars (e.g. SEM, SD) and the test used to calculate p-values in the respective figure legends. This information must be provided in the figure legends.

Please also include scale bars in all microscopy images.

When submitting your revised manuscript, please carefully review the instructions that follow below. Failure to include requested items will delay the evaluation of your revision.

1) a .docx formatted version of the manuscript text (including legends for main figures, EV figures and tables). Please make sure that the changes are highlighted to be clearly visible.

2) individual production quality figure files as .eps, .tif, .jpg (one file per figure). See https://wol-prod-cdn.literatumonline.com/pb-assets/embo-site/EMBOPress_Figure_Guidelines_061115-1561436025777.pdf for more info on how to prepare your figures.

3) We replaced Supplementary Information with Expanded View (EV) Figures and Tables that are collapsible/expandable online. A maximum of 5 EV Figures can be typeset. EV Figures should be cited as 'Figure EV1, Figure EV2" etc... in the text and their respective legends should be included in the main text after the legends of regular figures.

- For the figures that you do NOT wish to display as Expanded View figures, they should be bundled together with their legends in a single PDF file called *Appendix*, which should start with a short Table of Content. Appendix figures should be referred to in the main text as: "Appendix Figure S1, Appendix Figure S2" etc. See detailed instructions regarding expanded view here: <https://www.embopress.org/page/journal/14693178/authorguide#expandedview>

- Additional Tables/Datasets should be labeled and referred to as Table EV1, Dataset EV1, etc. Legends have to be provided in a separate tab in case of .xls files. Alternatively, the legend can be supplied as a separate text file (README) and zipped together with the Table/Dataset file.

4) a .docx formatted letter INCLUDING the reviewers' reports and your detailed point-by-point responses to their comments. As part of the EMBO Press transparent editorial process, the point-by-point response is part of the Review Process File (RPF), which will be published alongside your paper.

5) a complete author checklist, which you can download from our author guidelines <https://www.embopress.org/page/journal/14693178/authorguide>. Please insert information in the checklist that is also reflected in the manuscript. The completed author checklist will also be part of the RPF.

6) Please note that all corresponding authors are required to supply an ORCID ID for their name upon submission of a revised manuscript (<https://orcid.org/>). Please find instructions on how to link your ORCID ID to your account in our manuscript tracking system in our Author guidelines <https://www.embopress.org/page/journal/14693178/authorguide#authorshipguidelines>

7) We would also encourage you to include the source data for figure panels that show essential data. Numerical data should be provided as individual .xls or .csv files (including a tab describing the data). For blots or microscopy, uncropped images should be submitted (using a zip archive if multiple images need to be supplied for one panel). Additional information on source data and instruction on how to label the files are available at <https://www.embopress.org/page/journal/14693178/authorguide#sourcedata>.

We would also welcome the submission of cover suggestions, or motifs to be used by our Graphics Illustrator in designing a cover.

As part of the EMBO publication's Transparent Editorial Process, EMBO reports publishes online a Review Process File (RPF) to accompany accepted manuscripts. This File will be published in conjunction with your paper and will include the referee reports, your point-by-point response and all pertinent correspondence relating to the manuscript.

You are able to opt out of this by letting the editorial office know (emboreports@embo.org). If you do opt out, the Review Process File link will point to the following statement: "No Review Process

File is available with this article, as the authors have chosen not to make the review process public in this case."

I look forward to seeing a revised version of your manuscript when it is ready. Please let me know if you have questions or comments regarding the revision.

**REFEREE REPORTS**

**Referee #1:**

Here, Wolterhoff and colleagues show that PP2A is required for dendrite pruning of the class 4 multidendritic neurons in Drosophila. While PP2a was previously shown to be required for MT stability, they could not show genetic interactions with MT components that are known to be important in dendrite pruning and PP2A and therefore conclude that it likely functions via other routes. In contrast, they show that MICAL is likely downstream of PP2a as overexpression of MICAL suppresses PP2a induced pruning defects. In contrast to the accompanying paper from the Yu laboratory, they do not find that PP2a affects the expression of Sox14 and MICAL. Finally, they show that like MICAL, also overexpression of cofilin,another actin severing enzyme, also suppresses the PP2a induced pruning defects, strongly linking between PP2a and actin dynamics. They show that cofilin-GFP localization changes in KD of PP2a, thereby strengthening the link.

While this is an interesting manuscript, that tells a coherent story, I think that a few issues need to be addressed before publication. First, and most importantly, there are some important discrepancies between this groups' study and the Yu study. As presented, the data from the Yu group focusing on Sox14 and MICAL expression are stronger as they perform the experiments in mutant clones rather than RNAi. This should be investigated. Likewise the requirements of several subunits - most notably the regulatory units. Finally, even though this is a report, a brief discussion on the similarities as well as differences between the two studies is, in my opinion, important. Perhaps one can even propose an hypothesis that unifies at least most of the results.

* I think there is sometimes a confusing regarding the penetrance vs severity of the phenotypes. Please take care to be accurate here.

* as mentioned above, some experiments should be repeated with better reagents to figure out some of the discrepancies with other group.

* The structure of GFP-Cofilin are interesting but what do they mean? Has anyone shown that these are physiologically relevant structures? At the very least, you should show that this transgen also results in a suppression of the PP2a induced phenotype.

typos etc:
* "Once severed, pruning dendrites are subsequently fragmented in a caspase-dependent manner and phagocytosed by epidermal cells" - pruning should be pruned
* the prime symbols in the figures are upside down - perhaps this is a language issue but is confusing.

**Referee #2:**

The manuscript by Wolterhoff and colleagues describes the role of protein phosphatase PP2A in dendritic pruning in Drosophila c4da neurons. The authors focus on regulation of the actin cytoskeleton dynamics and identify the actin severing protein Mical as an important target of ecdysone signaling which is triggered during the onset of larvae metamorphosis. Although the findings are interesting and novel, the manuscript strongly lacks quantitative support for the statements, imaging data are of low quality, some of the assays are not appropriate and discussion is completely missing. Together, this limits my enthusiasm about the manuscript.

Major points:
1. At many instances, there is complete lack of quantification. For example, "knockdown of PP2A-

29B, encoding the A subunit of the serine/threonine phosphatase PP2A, caused a mild decrease in dendritic field coverage at the third instar larval stage (Fig.1 B)". Only single images are provided. Or "about half of the c4da neurons expressing dnMts showed a reduction in Mical levels (7/14) (Fig. 3 F)". Quantitative analysis of Mical immunofluorescence is needed to support this statement.
2. Image quality is low, many of them have a strong background and it is difficult to see what the authors want to show. I understand that they what to depict a larger field of view. Then showing traced images next to the originals will be helpful.
3. The authors mention 3 regulatory PP2A subunit downregulated via dsRNA or sgRNAs but only show data for Tws. The other 3 should be included into the supplement.
4. "Given that especially PP2A-29B knockdown yields highly penetrant pruning defects, we conclude that the observed genetic interactions between PP2A and Sox14/Mical do not reflect a role of PP2A in ecdysone-mediated gene expression." It is difficult to agree or disagree with this when there is no quantification. How about other factors that the ecdysone receptor is supposed to induce?
5. Overexpression of cofilin can induce many artifacts and has strong effects on neuronal morphology. More appropriate assays should be used to measure the actin turnover (i.e. FRAP).
6. Figure 4: higher magnification images of varicosities filled with lifeact::GFP should be added. Lifeact::GFP looks like a cell fill. Is it possible to quantify the intensity of F-actin vs regular volume marker?
7. Discussion is missing! Is it on purpose because there is nothing to discuss or just forgotten?

**Referee #3:**

The manuscript by Wolterhoff and colleagues on the role of PP2A during pruning has potential but also a number of weaknesses in its current form. As explained in greater detail below, the passage on regulatory subunits requires clear demonstration of the results available, the passage on microtubule regulators is over-rated, the statements about expression of Sox14 and Mical require quantification, and the hypothesis about actin spots should be made stronger through further genetic interactions including prominent actin regulators. To my taste, there needs to be a more elaborate discussion of results also in light of what is known about pruning and what this work conceptually adds to the field, ideally accompanied by a diagrammatic presentations of the hierarchical relationships found here. I recommend to reject the paper in its current state, but to provide the opportunity to re-submit since the principal findings are very interesting.

Detailed comments:

Introduction: make clearer where the introduction leads over into your presented work.

p.3: "The putative growth regulator headcase is also required for dendrite pruning [7]." Either explain or leave out

p4: "PP2A is a well-known cytoskeleton regulator with various targets linked to microtubule or F-actin dynamics" - name them, please

p6: Either show data for all regulatory subunits, or take out. What controls were done to prove that the conditional CRISPR was effective? Were RNAi experiments done in parallel?

p6/7: The section on MT regulation is not convincing. It presents some data on some known regulators, but is in no way comprehensive enough to raise a general statement about MT involvement. I suggest "MT regulators known to be involved in pruning seem not to act downstream of PP2A"

p7. Make clear to the reader whether Mical is up- or down-regulated during pruning. The paper requires pruning defect control data with knock-down of Mical (used later in the manuscript anyway) - even if published elsewhere, one needs to know how strong the effect is in the hands of these experimenters

p7: provide a brief explanation as to why DeltaCH might act as DN

p8: The lack of an effect demands further controls, especially since the DN approach for Mts might

not be appropriate. I would prefer to see repeats with independent LOF genetic tools for both genes.

p9: reduction observed upon tsr over-expression appears just to be significant. Please, provide p values so that the reader can make their own judgement. Sample numbers appear sufficiently high, especially for co-expression of S3A.

p11: I cannot follow the argumentation of "free actin"; in the presence of cofilin and Mical, there should be more G-actin but filamentous actin should be destabilised. Statements made by the authors should be changed or tested through genetic interaction with actin mutants -- although this will be tricky to interpret

p11: the passage on other kinases is weak since not supported by a strong and clear rationale. I would have preferred to see tests with other strong actin regulators such as profilin or enabled to support the actin patch hypothesis.

p12: I feel uneasy about being left alone without a proper discussion passage that sums it up for me, ideally accompanied by a diagram that illustrates the hierarchical relationships.

Figures: It would be helpful to add titles to graphs and also indicate the sample numbers in each bar. Are sample numbers single dendritic trees or ROIs? How many cells per animal? I do not understand the description "counting the number of neurons that still had dendrites attached to the soma, these data were analyzed using a two-tailed Fisher's exact test. To assess severity, we also counted the number of primary and secondary branches still attached to the soma at 18 h APF." In the latter case, do you count secondaries, or primaries with secondaries? All this needs to be made clear and transparent. -- indicate whether combinations for which there is no significance given are automatically non-significant? Otherwise, some important comparisons are missing in several graphs

Fig.2: G and H do not reflect the bar graph

Fig.3: readouts require some quantification. Especially the reduction of Mical in dnMTS looks strong. This would require a control with Mical knock-down.

Fig4: Description in D is incomplete; in the text varicosities are quantified. This should be shown here.

Fig5: remove bold from colour description, especially tomato is very hard to read; quantification should differentiate between somatic and dendritic for neurons with pruning defects.

1st Revision - authors' response 5 January 2020

**Referee #1:**

Here, Wolterhoff and colleagues show that PP2A is required for dendrite pruning of the class 4 multidendritic neurons in Drosophila. While PP2a was previously shown to be required for MT stability, they could not show genetic interactions with MT components that are known to be important in dendrite pruning and PP2A and therefore conclude that it likely functions via other routes. In contrast, they show that MICAL is likely downstream of PP2a as overexpression of MICAL suppresses PP2a induced pruning defects. In contrast to the accompanying paper from the Yu laboratory, they do not find that PP2a affects the expression of Sox14 and MICAL. Finally, they show that like MICAL, also overexpression of cofilin,another actin severing enzyme, also suppresses the PP2a induced pruning defects, strongly linking between PP2a and actin dynamics. They show that cofilin-GFP localization changes in KD of PP2a, thereby strengthening the link.

While this is an interesting manuscript, that tells a coherent story, I think that a few issues need to be addressed before publication. First, and most importantly, there are some important discrepancies between this groups' study and the Yu study. As presented, the data from the Yu group focusing on Sox14 and MICAL expression are stronger as they perform the experiments in mutant clones rather

than RNAi. This should be investigated. Likewise the requirements of several subunits - most notably the regulatory units. Finally, even though this is a report, a brief discussion on the similarities as well as differences between the two studies is, in my opinion, important. Perhaps one can even propose an hypothesis that unifies at least most of the results.

Our response:
Thank you for these comments. We have now addressed the issue of Mical expression. It turns out that at least Mical expression is slightly delayed upon loss of PP2A. We missed this effect because we usually perform our Mical stainings at 2 h APF rather than at the WP stage. At the WP stage, Mical expression is indeed reduced. However, this effect is not as pronounced as in other mutants with Mical expression defects (e. g., vcp or eIF4A). We therefore think that it cannot be the only cause for the observed genetic interactions.

* I think there is sometimes a confusing regarding the penetrance vs severity of the phenotypes. Please take care to be accurate here.

Our response:
We judge our pruning phenotypes by two criteria. Firstly, we ask what percentage of the manipulated neurons show the phenotype (penetrance), and secondly, we ask how severe the phenotype is. Towards this end, we take the sum of the number of primary and secondary dendrites as a measure. We now explain this explicitly in the Results section (p. 5) and use the same terms ("penetrant" and "severe") throughout. We hope this clarifies our approach.

* as mentioned above, some experiments should be repeated with better reagents to figure out some of the discrepancies with other group.

Our response:
It turns out that the discrepancy was mostly in the slightly different timepoints used by us and the Yu lab, not the reagents (see above).

* The structure of GFP-Cofilin are interesting but what do they mean? Has anyone shown that these are physiologically relevant structures? At the very least, you should show that this transgen also results in a suppression of the PP2a induced phenotype.

Our response:
Thanks for this comment. In the revised manuscript, we tried to characterize the GFP::tsr transgene better. Firstly, we show that GFP::tsr does ameliorate the pruning defects caused by PP2A-29B knockdown (new Figure 5 F, N). The suppression is not as strong as with the untagged versions, but this is likely in part due to expression strength. In our hands, UAS transgenes inserted into attP sites are not as strong as randomly inserted transgenes made with the older delta23 transposase method. Active cofilin is known to form punctae that contain actin ("actin rods"), especially when overexpressed. Therefore, we cannot entirely guarantee that the observed structures are physiological. However, as GFP::tsr also localizes to the actin-rich spikes of c3da neurons (new Figure EV 5), we think that it can serve as a proxy for actin dynamics. This would best explain the effects of Mical and PP2A knockdown.
We should say here that we have tried to generate other actin reporters, including an actin-Dendra for photoconversion and dynamics measurements. These tools have not given us interpretable results. In fact, a local actin expert told us that actin variants with large tags are only incorporated into F-actin at very low rates, and that the search for a good actin dynamics marker is still ongoing in the actin field. We hope that the combination of markers and genetics still provides decent-enough evidence for our hypothesis.

typos etc:
* "Once severed, pruning dendrites are subsequently fragmented in a caspase-dependent manner and phagocytosed by epidermal cells" - pruning should be pruned
* the prime symbols in the figures are upside down - perhaps this is a language issue but is confusing.

Our response:
Thanks – we changed this.

**Referee #2:**

The manuscript by Wolterhoff and colleagues describes the role of protein phosphatase PP2A in dendritic pruning in Drosophila c4da neurons. The authors focus on regulation of the actin cytoskeleton dynamics and identify the actin severing protein Mical as an important target of ecdysone signaling which is triggered during the onset of larvae metamorphosis. Although the findings are interesting and novel, the manuscript strongly lacks quantitative support for the statements, imaging data are of low quality, some of the assays are not appropriate and discussion is completely missing. Together, this limits my enthusiasm about the manuscript.

Major points:
1. At many instances, there is complete lack of quantification. For example, "knockdown of PP2A-29B, encoding the A subunit of the serine/threonine phosphatase PP2A, caused a mild decrease in dendritic field coverage at the third instar larval stage (Fig.1 B)". Only single images are provided. Or "about half of the c4da neurons expressing dnMts showed a reduction in Mical levels (7/14) (Fig. 3 F)". Quantitative analysis of Mical immunofluorescence is needed to support this statement.

Our response:
Thank you for this comment. In order to address concerns regarding quantification, we added a quantitative assessment of the effects of loss of PP2A on larval dendrite elaboration (new Figure EV1). We added N and P values in all graphs (Figures 1 – 5) and quantified the Mical stainings in Figure 3.

2. Image quality is low, many of them have a strong background and it is difficult to see what the authors want to show. I understand that they what to depict a larger field of view. Then showing traced images next to the originals will be helpful.

Our response:
We tried to address this by carefully adjusting the brightness in some of the darker images. We hope that the observed effects can now be seen better.

3. The authors mention 3 regulatory PP2A subunit downregulated via dsRNA or sgRNAs but only show data for Tws. The other 3 should be included into the supplement.

Our response:
We now added our complete CRISPR/RNAi/mutant analyses for the B/B' subunits Tws, Wrd, PR72, and Wdb. Our data now show that Wdb is also required for dendrite pruning, and that Tws and Wdb likely act in a redundant manner.

4. "Given that especially PP2A-29B knockdown yields highly penetrant pruning defects, we conclude that the observed genetic interactions between PP2A and Sox14/Mical do not reflect a role of PP2A in ecdysone-mediated gene expression." It is difficult to agree or disagree with this when there is no quantification. How about other factors that the ecdysone receptor is supposed to induce?

Our response:
We have now addressed the issue of Mical expression by quantification and by looking at other developmental timepoints. It turns out that at least Mical expression is slightly delayed upon loss of PP2A. We missed this effect because we usually perform our Mical stainings at 2 h APF rather than at the WP stage. At the WP stage, Mical expression is indeed reduced. However, this effect is not as

pronounced as in other mutants with Mical expression defects (e. g., vcp or eIF4A). We therefore think that it cannot be the only cause for the observed genetic interactions.

5. Overexpression of cofilin can induce many artifacts and has strong effects on neuronal morphology. More appropriate assays should be used to measure the actin turnover (i.e. FRAP).

Our response:
As mentioned in the response to reviewer 1, we have tried to generate other actin reporters, such as actin-Dendra for photoconversion and dynamics measurements but these tools did not give clear interpretable results. In fact, a local actin expert told us that actin variants with large tags are only incorporated into F-actin at very low rates, and that the search for a good actin dynamics marker is still ongoing in the actin field.
We therefore tried to charaterize GFP::tsr in other ways. Firstly, we show that it also partially suppresses PP2A-29B knockdown (new Fig. 5 N). Furthermore, we provide images of larval c4da neuron morphology upon GFP::tsr expression which looks overall normal (Fig. EV 5). In addition, we performed genetic interaction tests and found that PP2A interacts with other actin regulators such as chicadee/profilin and moesin. We think that the combination of these assays provides additional support for our hypothesis.

6. Figure 4: higher magnification images of varicosities filled with lifeact::GFP should be added. Lifeact::GFP looks like a cell fill. Is it possible to quantify the intensity of F-actin vs regular volume marker?

Our response:
Thank you for this suggestion. We added the higher magnification image as an inset in the figure (now Figure 5 C). We tried to normalize the lifeact::GFP to tdtomato in these images. Since the varicosities are quite big, the tdtomato signal there is also stronger and the result was not significant. We therefore toned down our interpretation that the varicosities are actin-rich.

7. Discussion is missing! Is it on purpose because there is nothing to discuss or just forgotten?

Our response:
We added a Discussion section where we discuss our data in relation to the Yu lab study (we think it is nicely complementary) as well as the nature of the GFP::cofilin localization.

**Referee #3:**

The manuscript by Wolterhoff and colleagues on the role of PP2A during pruning has potential but also a number of weaknesses in its current form. As explained in greater detail below, the passage on regulatory subunits requires clear demonstration of the results available, the passage on microtubule regulators is over-rated, the statements about expression of Sox14 and Mical require quantification, and the hypothesis about actin spots should be made stronger through further genetic interactions including prominent actin regulators. To my taste, there needs to be a more elaborate discussion of results also in light of what is known about pruning and what this work conceptually adds to the field, ideally accompanied by a diagrammatic presentations of the hierarchical relationships found here. I recommend to reject the paper in its current state, but to provide the opportunity to re-submit since the principal findings are very interesting.

Detailed comments:

Introduction: make clearer where the introduction leads over into your presented work.

Our response:
We now state at the beginning of the corresponding paragraph: “In this study, we investigated the role of the serine/threonine phosphatase PP2A in c4da neurons and found that it is required for c4da

neuron dendrite pruning." (p. 4)

p.3: "The putative growth regulator headcase is also required for dendrite pruning [7]." Either explain or leave out

Our response:
We now explain better that hdc is also an ecdysone target.

p4: "PP2A is a well-known cytoskeleton regulator with various targets linked to microtubule or F-actin dynamics" - name them, please

Our response:
We now added specific references for cytoskeletal regulators as PP2A targets (tau as a MT regulator, a Rho GEF, and, indirectly, ERM domain proteins.

p6: Either show data for all regulatory subunits, or take out. What controls were done to prove that the conditional CRISPR was effective? Were RNAi experiments done in parallel?

Our response:
In order to better characterize the PP2A holoenzyme required for dendrite pruning, we have added a MARCM analysis of the catalytic subunit Mts (Figure 1) and extensive RNAi and/or mutant analyses of the B subunits which lead us to conclude that the subunits Twins and Widerborst regulate pruning in a redundant manner (new Figure EV2).

p6/7: The section on MT regulation is not convincing. It presents some data on some known regulators, but is in no way comprehensive enough to raise a general statement about MT involvement. I suggest "MT regulators known to be involved in pruning seem not to act downstream of PP2A"

Our response:
We agree that our previous phrasing was too broad. We therefore rephrased and now state that PP2A does not regulate pruning via the Par-1/tau pathway.

p7. Make clear to the reader whether Mical is up- or down-regulated during pruning. The paper requires pruning defect control data with knock-down of Mical (used later in the manuscript anyway) - even if published elsewhere, one needs to know how strong the effect is in the hands of these experimenters

Our response:
We added an experiment to confirm that Mical knockdown causes strong dendrite pruning defects (new Figure EV 5).

p7: provide a brief explanation as to why DeltaCH might act as DN

Our response:
We added the following sentence in the corresponding paragraph: " ...overexpression of *MicalΔCH* in c4da neurons alone had a dominant effect and caused strong dendrite pruning defects (Fig. 2 E, K, L), possibly indicating that Mical must act as a dimer, or that the CH domain is required for substrate release."

p8: The lack of an effect demands further controls, especially since the DN approach for Mts might not be appropriate. I would prefer to see repeats with independent LOF genetic tools for both genes.

Our response:
We have now addressed the issue of Mical expression by quantification and by looking at other developmental timepoints. It turns out that at least Mical expression is delayed upon PP2A knockdown. We missed this effect because we usually perform our Mical stainings at 2 h APF rather than at the WP stage. At the WP stage, Mical expression is indeed reduced by PP2A knockdown. However, this effect is not as pronounced as in other mutants with Mical expression defects (e. g., vcp or eIF4A). We therefore think that it cannot be the only cause for the observed genetic interactions.

p9: reduction observed upon tsr over-expression appears just to be significant. Please, provide p values so that the reader can make their own judgement. Sample numbers appear sufficiently high, especially for co-expression of S3A.

Our response:
We provided the exact P values in the corresponding figures.

p11: I cannot follow the argumentation of "free actin"; in the presence of cofilin and Mical, there should be more G-actin but filamentous actin should be destabilised. Statements made by the authors should be changed or tested through genetic interaction with actin mutants -- although this will be tricky to interpret

Our response:
We have now added a Discussion including a paragraph on the significance of the GFP-cofilin dots (p. 14). As in particular Mical dsRNA suppresses the decrease in cofilin dots at the onset of the pupal stage, we think that they reflect an aspect of actin dynamics in the neurons.

p11: the passage on other kinases is weak since not supported by a strong and clear rationale. I would have preferred to see tests with other strong actin regulators such as profilin or enabled to support the actin patch hypothesis.

Our response:
We have now added genetic interactions with actin regulators including ena and profilin (new Figure 5). The rationale for the kinase experiment is that PP2A must counteract a phosphorylation event (since PP2A holoenzyme is involved).

p12: I feel uneasy about being left alone without a proper discussion passage that sums it up for me, ideally accompanied by a diagram that illustrates the hierarchical relationships.

Our response:
We have now added a schematic image of how we think PP2A regulates pruning (new Figure 6).

Figures: It would be helpful to add titles to graphs and also indicate the sample numbers in each bar. Are sample numbers single dendritic trees or ROIs? How many cells per animal? I do not understand the description "counting the number of neurons that still had dendrites attached to the soma, these data were analyzed using a two-tailed Fisher's exact test. To assess severity, we also counted the number of primary and secondary branches still attached to the soma at 18 h APF." In the latter case, do you count secondaries, or primaries with secondaries? All this needs to be made clear and transparent. -- indicate whether combinations for which there is no significance given are automatically non-significant? Otherwise, some important comparisons are missing in several graphs

Our response:
We added the total number of neurons for each group in each graph (that is always the N in our experiments). We also explained the procedure in more detail in the Materials and Methods section and explained our quantification in the main text when in comes up first.

Fig.2: G and H do not reflect the bar graph

Our response:
We replaced the panels with images that better reflect the mean values.

Fig.3: readouts require some quantification. Especially the reduction of Mical in dnMTS looks strong. This would require a control with Mical knock-down.

Our response:
We have now addressed the issue of Mical expression by quantification and by looking at other developmental timepoints. It turns out that at least Mical expression is delayed upon PP2A loss.

Fig4: Description in D is incomplete; in the text varicosities are quantified. This should be shown here.

Our response:
We now explained our quantification in more detail in the corresponding figure legend.

Fig5: remove bold from colour description, especially tomato is very hard to read; quantification should differentiate between somatic and dendritic for neurons with pruning defects.

Our response:
We changed the corresponding figure (now Figure 4) and added images showing stretches of dendrites expressing GFP::tsr in Figure EV5.

2nd Editorial Decision 12 February 2020

Thank you for the submission of your revised manuscript. We have now received the enclosed comments from all referees. As you will see, the referees ask for some more clarifications, but overall all agree that the study can be published.
I would thus like to invite you to address all remaining concerns, as well as the points below, so that we can proceed with the official acceptance of your work. Please also submit a point-by-point response to the last concerns with your final manuscript.

The reference style needs to be corrected, not more than 10 authors should be listed before "et al".

Please list the figure panels in sequential order in the manuscript text. Fig 5M and Fig 6 callouts are missing.

The author name needs to be removed from the figure files.

Fig 2C and Fig EV 3E seem to be identical. Please correct/clarify.

For Figs EV1+3+5 you added "Numbers of analyzed neurons are given in the graph" to the legend. Please also specify whether or not the numbers are derived from at least 3 independent experiments.

I attach to this email a manuscript word file with comments by our data editors, all tracked. Please address all comments in the final manuscript file.

EMBO press papers are accompanied online by A) a short (1-2 sentences) summary of the findings and their significance, B) 2-3 bullet points highlighting key results and C) a synopsis image that is 550x200-400 pixels large (the height is variable). You can either show a model or key data in the

synopsis image. Please note that text needs to be readable at the final size. Please send us this information along with the revised manuscript.

Please make sure that the back to back paper from Yu's group is cited and included in the reference list. I could not find it now.

I look forward to seeing a final version of your manuscript as soon as possible.
Please let me know if you have any questions or comments.

**REFEREE REPORTS**

**Referee #1:**

In this revised manuscript, the Rumpf group have addressed the vast majority of my concerns. I think it is nice that their work now is agrees more with the Yu group, despite some differences. I am also glad that they added a discussion, especially to discuss those differences between the two groups. All in all, this study, alone and even moreso alongside the Yu manuscript, provide an important advance on how pp2a regulates dendrite pruning by at least two parallel pathways. The interplay between actin and MT dynamics and how it affect pruning remains to be further elucidated.

**Referee #2:**

The manuscript by Wolterhoff et al has significantly improved in the revised version but there are still some inconsistencies, missing controls and technical/editorial issues.

1. Figure 1: There is still missing field coverage quantification for C, D, E and F. Same in the Figure 2. This quantification is important for later interpretation of the results.

2. Figure 3: The large variability from only n=3 makes the Sox14 quantification statement on whether Sox14 levels are changed upon PP2A-29B knockdown impossible to determine. In the initial submission, the authors reported "about half of the c4da neurons expressing dnMts showed a reduction in Mical levels (7/14) (Fig. 3 F)". Yet the reported n in Fig3I for dnMts is now 12. Why some samples are not included in the analysis anymore? Fig3E-F + I The time point for the control was 2h APF, so there should be a 0h APF / assessment of the control for a proper comparison with the 0h APF PP2A-29B knockdown.

3. The authors write "Given that especially PP2A-29B knockdown yields highly penetrant pruning defects under conditions of relatively normal Mical expression, we conclude that the observed genetic interactions between PP2A and Sox14/Mical cannot be explained through a role of PP2A in Mical gene expression alone." This is why the field coverage issue is so important to quantify, it is possible that there are also defects occurring before pruning.

4. "In contrast to control neurons, GFP::tsr was evenly dispersed in larval c4da neurons expressing PP2A-29B dsRNA, with no punctae visible (Fig. 4 L, M)." This could point to the defects before pruning. Perhaps pruning in many PP2A-29B knockout neurons starts earlier?

5. "Pruning dendrites of c4da neurons develop varicosities as a sign of degeneration during the early pruning stages between approximately 3 - 12 h APF [10]." Regular varicosities in control neurons would be shown to see whether the on-set is similar between both.

6. Figure 6 is not referenced in the text.

7. Figure EV1: the n-numbers are very different from those in Figure 1.

8. Figure EV3 and 6 should be included in the results session, they only appear in Discussion.

9. Statistics are not always consistent. Please add not only total number of neurons but also number

of flies in all figures.

10. "This suppression wa slower than with untagged tsr," Typo: "was". Also using term "slower" is not fully correct here. Perhaps rephrase: "This suppression was reduced compared to untagged tsr."?

**Referee #3:**

Overall, the data have now improved as suggested in my last report, and there are only minor details (see below). However, I still feel that the authors should strengthen their argumentation. In their hands, microtubule manipulations have little effect and the conclusion of these experiments has been toned down appropriately. Far more important is the fact that there are now more data with different actin regulators which all indicate a role of PP2A in mediating dendrite pruning through destabilising actin networks. This has to be made clearer: The title, suggests as a novelty that PP2A is required for dendrite pruning which, according to the introduction, has been clear from previous work on Tws and Wdb. The focus should therefore rather lie on the role of actin in mediating this effect. This should be emphasised and made clear in the second half of the abstract, at the end of the introduction and through a more focussed re-write of the last two results sections (see comments below). Also in the discussion, the different findings are currently being discussed in the same sequence as they occur in the results part, with almost equal emphasis, and actin being pushed into the second half rather than presenting as the key focus of the discussion. In conclusion, I would recommend publication but would urge the authors to invest some additional time to improve the message, hece reach of their paper.

Detailed comments

The second half of the introduction is confused and makes little sense without having read the whole paper. At least four factors (including ecdyson) are named, but how they relate to each other remains unclear. The message should come across strongly here.

last para of intro: It would be good to let the reader know that PP2A was picked in a screen for pruning regulators. Or do I misunderstand this at the beginning of the results part? Was PP2A knocked down as a prime candidate? Please, explain your rationale.

p.4, last section: mention Mts as a C subunit since it will play a role in this manuscript, and also Tws as a B subunit

p.4, last para: add reference for Tws and Wdb roles in pruning

Conclusions at the end of the introduction are too vague for my taste; there is no real take-home message, especially when considering that the authors say two sentences before that PP2A is involved in pruning. Was the problem that involvement of PP2A was known but not through which mechanism?

p.5: What is Orco? Why was it used as a control?

p.7: The rationale for the pruning section reads unnecessarily complex. In the intro, the authors state that PP2A is known to regulate both actin and MT regulators. Why not state this here, followed by a sentence saying that they started by looking first into candidate MT regulators.

P.8, 1st para: explain what role the CH domain plays in dimerisation and how lack of dimerisation might cause a dominant effect?

Fig.3: in all panels indicate the developmental time as done in panels G and H. I wish to see a bar for Orco controls at both time points: WP and 2h APF. Please, remind the reader at what timepoint the actual pruning takes place. Hence, what difference would a 2hr delay of Mical upregulation mean?

p.10, last para: the cofilin accumulations look almost pathological and ideally should be co-stained for actin; if actin co-aggregates but similar aggregates are not found in controls without cofilin

overexpression, then such actin accumulations would be suggested to be induced by cofilin, reminding of actin/cofilin co-assembled aggregations in Alzheimer's: Bamburg & Bloom, 2009, Cell Motil Cytoskeleton 66, 635ff. -- http://www.ncbi.nlm.nih.gov/entrez/query.fcgi?cmd=Retrieve&db=PubMed&dopt=Citation&list_uids=19479823

Fig.EV5: indicate what proteins GFP and tomato are linked to. I absolutely fail to see any actin accumulations in EV5 C and actin seems not even to be shown.

p.11/12: What point do the authors want to make with the varicosity data? Should they not better be moved as an additional observation into the first section of this results part? Here it interrupts the flow of the argument in my view.

The last two sections lack a simple structure and should ideally be rewritten to make a clear point along the lines of: Genetic interaction with various actin regulators strongly suggests PP2A to act through actin regulation in dendrite pruning".

2nd Revision - authors' response 27 February 2020

**Referee 1:**

In this revised manuscript, the Rumpf group have addressed the vast majority of my concerns. I think it is nice that their work now is agrees more with the Yu group, despite some differences. I am also glad that they added a discussion, especially to discuss those differences between the two groups. All in all, this study, alone and even moreso alongside the Yu manuscript, provide an important advance on how pp2a regulates dendrite pruning by at least two parallel pathways. The interplay between actin and MT dynamics and how it affect pruning remains to be further elucidated.

Our response:
Thanks!

**Referee 2:**

The manuscript by Wolterhoff et al has significantly improved in the revised version but there are still some inconsistencies, missing controls and technical/editorial issues.

1. Figure 1: There is still missing field coverage quantification for C, D, E and F. Same in the Figure 2. This quantification is important for later interpretation of the results.

Our response:
We added coverage information for PP2A MARCM, dnMts, and mts MARCM (new Figure EV1 D). These results confirm our (and previously other people's) notion that loss of PP2A causes defects in dendrite morphogenesis. We also addressed whether these defects can be rescued by overexpression of Mical, but this does not seem to be the case (new Figure EV1 E - G).

2. Figure 3: The large variability from only n=3 makes the Sox14 quantification statement on whether Sox14 levels are changed upon PP2A-29B knockdown impossible to determine. In the initial submission, the authors reported "about half of the c4da neurons expressing dnMts showed a reduction in Mical levels (7/14) (Fig. 3 F)". Yet the reported n in Fig3I for dnMts is now 12. Why some samples are not included in the analysis anymore? Fig3E-F + I The time point for the control was 2h APF, so there should be a 0h APF / assessment of the control for a proper comparison with the 0h APF PP2A-29B knockdown.

Our response:

We performed additional Sox14 staining experiments to increase the N for control and PP2A knockdown samples and updated Figure 3 accordingly (new Figure 3). The data now in fact indicate that neurons lacking PP2A have slightly higher levels of Sox14. We have seen qualitatively similar effects in other mutants with pruning defects and we think this might reflect a feedback upregulation. Again, the seeming discrepancy with the Yu lab data is probably caused by the later timepoint at which we are looking.
The difference in the sample number for the Mical stainings in the dnMts background stems from the fact that two samples were not suitable for quantitative analysis, i. e., while we could qualitatively say that Mical was (or was not) expressed, they lacked the adjacent c1da neuron in the same image that we use for fluorescence normalization.
We also added Mical stainings in Orco dsRNA controls at 0 h APF as control for the corresponding PP2A knockdown (new Figure 3). This experiment basically supports our notion that lack of PP2A leads to a delay in Mical expression early on at the wihite pupal stage, but not at 2 h APF when we usually perform our experiments.

3. The authors write "Given that especially PP2A-29B knockdown yields highly penetrant pruning defects under conditions of relatively normal Mical expression, we conclude that the observed genetic interactions between PP2A and Sox14/Mical cannot be explained through a role of PP2A in Mical gene expression alone." This is why the field coverage issue is so important to quantify, it is possible that there are also defects occurring before pruning.

Our response:
Thank you for this comment. Our data indicate that a role of PP2A in actin regulation is the cause for the observed pruning defects. As we pointed out, a role for PP2A (precisely, the PP2A B subunit Widerborst) in larval dendrite morphogenesis has previously been described (Das et al., 2017, Genetics). That study also linked PP2A to actin regulation during dendrite morphogenesis. It is indeed very interesting to speculate that the defects in larval dendrite morphology are due to the same actin regulatory pathway as the defects we observed during dendrite pruning. We therefore added the following sentence in the discussion: „Since the data presented by us and others [23, 24] suggest that loss of PP2A affects actin dynamics and hence possibly both F-actin assembly and disassembly, it is interesting to speculate that the underlying causes for the larval dendrite morphogenesis defects and the pruning defects may be similar." (p. 14)

4. "In contrast to control neurons, GFP::tsr was evenly dispersed in larval c4da neurons expressing PP2A-29B dsRNA, with no punctae visible (Fig. 4 L, M)." This could point to the defects before pruning. Perhaps pruning in many PP2A-29B knockout neurons starts earlier?

Our response:
Please see our response under point 3.

5. "Pruning dendrites of c4da neurons develop varicosities as a sign of degeneration during the early pruning stages between approximately 3 - 12 h APF [10]." Regular varicosities in control neurons would be shown to see whether the on-set is similar between both.

Our response:
We referred here to the normally occurring varicosities and to those in neurons lacking Par-1. To make the distinction clearer, we now denote a "normal" varicosity in the image of the neuron lacking Par-1 (Fig. EV 4B).

6. Figure 6 is not referenced in the text.

Our response:
Thanks for pointing this out. We introduced the following additional sentence in the discussion: „We therefore tentatively place PP2A in a pathway at least partially in parallel to Mical during dendrite pruning (Fig. 6)." (p. 14)

7. Figure EV1: the n-numbers are very different from those in Figure 1.

Our response:
Yes, this is a different data set that we generated for the analysis of larval dendrite morphology. The quantifications shown in Figure 1 refer to the pruning defects.

8. Figure EV3 and 6 should be included in the results session, they only appear in Discussion.

Our response:
We now describe the kinase results (Figure EV6) in the results section. Figure EV3 is described there already (lack of genetic interactions between PP2a and PAR-1/tau pathway).

9. Statistics are not always consistent. Please add not only total number of neurons but also number of flies in all figures.

Our response:
We now added the number of neurons and from how many flies they are derived in all figures.

10. "This suppression wa slower than with untagged tsr," Typo: "was". Also using term "slower" is not fully correct here. Perhaps rephrase: "This suppression was reduced compared to untagged tsr."?

Our response:
Thanks for catching the typo. It is corrected now.

**Referee 3:**

Overall, the data have now improved as suggested in my last report, and there are only minor details (see below). However, I still feel that the authors should strengthen their argumentation. In their hands, microtubule manipulations have little effect and the conclusion of these experiments has been toned down appropriately. Far more important is the fact that there are now more data with different actin regulators which all indicate a role of PP2A in mediating dendrite pruning through destabilising actin networks. This has to be made clearer: The title, suggests as a novelty that PP2A is required for dendrite pruning which, according to the introduction, has been clear from previous work on Tws and Wdb. The focus should therefore rather lie on the role of actin in mediating this effect. This should be emphasised and made clear in the second half of the abstract, at the end of the introduction and through a more focussed re-write of the last two results sections (see comment s below). Also in the discussion, the different findings are currently being discussed in the same sequence as they occur in the results part, with almost equal emphasis, and actin being pushed into the second half rather than presenting as the key focus of the discussion. In conclusion, I would recommend publication but would urge the authors to invest some additional time to improve the message, hece reach of their paper.

Our response:
Thanks for these comments. There seems to be a misunderstanding in part – the novelty of our report is twofold, firstly it was not known that PP2A (any subunit) is required for dendrite pruning, and secondly, we present a lot of evidence for actin regulation by PP2A during dendrite pruning, when the role of actin during dendrite pruning had previously been neglected. What was previously known is that some PP2A subunits can regulate actin in c4da neurons at other stages of development.
In order to clarify these connections, we have reorganized and rewritten the introduction, stating both that we newly identified PP2A as a pruning regulator and that very little was known about actin regulation during dendrite pruning.

Detailed comments

The second half of the introduction is confused and makes little sense without having read the whole paper. At least four factors (including ecdyson) are named, but how they relate to each other remains unclear. The message should come across strongly here.

Our response:
Thanks for this comment. We rewrote this section and tried to explain this better what was known and what is novel about our findings.

last para of intro: It would be good to let the reader know that PP2A was picked in a screen for pruning regulators. Or do I misunderstand this at the beginning of the results part? Was PP2A knocked down as a prime candidate? Please, explain your rationale.

Our response:
We introduced the following sentence in the introduction: "In a survey of phosphoregulators of dendrite pruning, we found that loss of the scaffold subunit PP2A-29B caused strong dendrite pruning defects."

p.4, last section: mention Mts as a C subunit since it will play a role in this manuscript, and also Tws as a B subunit

Our response:
We now explicitly mention that mts encodes the PP2A catalytic subunit.

p.4, last para: add reference for Tws and Wdb roles in pruning

Our response:
This is a misunderstanding – a role for Tws and Wdb was not previously known. It was only known that Wdb and Tws can regulate actin-related processes in neurons. We have included references for regulation of actin-related factors by PP2A (refs. 20, 24). We hope this is clearer in the revised text.

Conclusions at the end of the introduction are too vague for my taste; there is no real take-home message, especially when considering that the authors say two sentences before that PP2A is involved in pruning. Was the problem that involvement of PP2A was known but not through which mechanism?

Our response:
Thanks for this comment. We rewrote this section and tried to explain this better what was known and what is novel about our findings.

p.5: What is Orco? Why was it used as a control?

Our response:
This is Odorant receptor co-receptor, known to be only expressed in olfactory neurons. We added an explanation (p.5).

p.7: The rationale for the pruning section reads unnecessarily complex. In the intro, the authors state that PP2A is known to regulate both actin and MT regulators. Why not state this here, followed by a sentence saying that they started by looking first into candidate MT regulators.

Our response:
We shortened this section accordingly.

© European Molecular Biology Organization

P.8, 1st para: explain what role the CH domain plays in dimerisation and how lack of dimerisation might cause a dominant effect?

Our response:
This is of course speculative – the idea was that an inactive molecule in a multimeric enzyme complex could inhibit the activity of the whole complex. As the multimeric state of Mical is not known, we rephrased this section: "...overexpression of *MicalΔCH* in c4da neurons alone had a dominant effect and caused strong dendrite pruning defects (Fig. 2 E, K, L), possibly reflecting a direct involvement of this domain in the catalytic cycle of Mical."

Fig.3: in all panels indicate the developmental time as done in panels G and H. I wish to see a bar for Orco controls at both time points: WP and 2h APF. Please, remind the reader at what timepoint the actual pruning takes place. Hence, what difference would a 2hr delay of Mical upregulation mean?

Our response:
We added the 0 h APF Orco control (new panel 3H) and the quantification. We also indicate the developmental timepoints in every panel now. In order to better explain our interpretation of these results, we rephrased the corresponding section: " We interpret these data as indicating a delay in Mical expression upon PP2A downregulation. However, dendrite severing and pruning takes places between 5 - 10 h APF and we assess pruning at 18 h APF, and the pruning defects induced by *PP2A-29B* knockdown are still highly penetrant and severe even at this latter stage. We therefore conclude that the observed genetic interactions between PP2A and Sox14/Mical cannot be explained through a role of PP2A in Mical gene expression alone."

p.10, last para: the cofilin accumulations look almost pathological and ideally should be co-stained for actin; if actin co-aggregates but similar aggregates are not found in controls without cofilin overexpression, then such actin accumulations would be suggested to be induced by cofilin, reminding of actin/cofilin co-assembled aggregations in Alzheimer's: Bamburg & Bloom, 2009, Cell Motil Cytoskeleton 66, 635ff. -- http://www.ncbi.nlm.nih.gov/entrez/query.fcgi?cmd=Retrieve&db=PubMed&dopt=Citation&list_uids=19479823

Our response:
Unfortunately it is very difficult to stain actin in this tissue. But the localization of GFP::tsr with c3da dendritic spikes is a very good proxy here (see below). We also speculate that the punctae contain actin and inserted the suggested reference. Thanks!

Fig.EV5: indicate what proteins GFP and tomato are linked to. I absolutely fail to see any actin accumulations in EV5 C and actin seems not even to be shown.

Our response:
This is actually known from previous work – we had forgotten to cite this appropriately. This is now corrected, thanks. We also indicate now that the green channel shows GFP::tsr.

p.11/12: What point do the authors want to make with the varicosity data? Should they not better be moved as an additional observation into the first section of this results part? Here it interrupts the flow of the argument in my view.

Our response:
Thank you for this suggestion. We changed the order of the manuscript and display the varicosity data as Fig. EV4 now, where we look at potential links between PP2A, PAR-1 and microtubules. We agree that this improves the flow of the argument.

The last two sections lack a simple structure and should ideally be rewritten to make a clear point along the lines of: Genetic interaction with various actin regulators strongly suggests PP2A to act through actin regulation in dendrite pruning".

Our response:
See previous response.

Accepted 4 March 2020

I am very pleased to accept your manuscript for publication in the next available issue of EMBO reports. Thank you for your contribution to our journal.

© European Molecular Biology Organization 

EMBO PRESS

YOU MUST COMPLETE ALL CELLS WITH A PINK BACKGROUND ➔

PLEASE NOTE THAT THIS CHECKLIST WILL BE PUBLISHED ALONGSIDE YOUR PAPER

Corresponding Author Name: Sebastian Rumpf

Journal Submitted to: EMBO Reports
Manuscript Number: EMBOR-2019-48870V2

## Reporting Checklist For Life Sciences Articles (Rev. June 2017)

This checklist is used to ensure good reporting standards and to improve the reproducibility of published results. These guidelines are consistent with the Principles and Guidelines for Reporting Preclinical Research issued by the NIH in 2014. Please follow the journal's authorship guidelines in preparing your manuscript.

### A- Figures
### 1. Data

**The data shown in figures should satisfy the following conditions:**

- the data were obtained and processed according to the field's best practice and are presented to reflect the results of the experiments in an accurate and unbiased manner.
- figure panels include only data points, measurements or observations that can be compared to each other in a scientifically meaningful way.
- graphs include clearly labeled error bars for independent experiments and sample sizes. Unless justified, error bars should not be shown for technical replicates.
- if n< 5, the individual data points from each experiment should be plotted and any statistical test employed should be justified
- Source Data should be included to report the data underlying graphs. Please follow the guidelines set out in the author ship guidelines on Data Presentation.

### 2. Captions

**Each figure caption should contain the following information, for each panel where they are relevant:**

- a specification of the experimental system investigated (eg cell line, species name).
- the assay(s) and method(s) used to carry out the reported observations and measurements
- an explicit mention of the biological and chemical entity(ies) that are being measured.
- an explicit mention of the biological and chemical entity(ies) that are altered/varied/perturbed in a controlled manner.

- the exact sample size (n) for each experimental group/condition, given as a number, not a range;
- a description of the sample collection allowing the reader to understand whether the samples represent technical or biological replicates (including how many animals, litters, cultures, etc.).
- a statement of how many times the experiment shown was independently replicated in the laboratory.
- definitions of statistical methods and measures:
  - common tests, such as t-test (please specify whether paired vs. unpaired), simple χ2 tests, Wilcoxon and Mann-Whitney tests, can be unambiguously identified by name only, but more complex techniques should be described in the methods section;
  - are tests one-sided or two-sided?
  - are there adjustments for multiple comparisons?
  - exact statistical test results, e.g., P values = x but not P values < x;
  - definition of 'center values' as median or average;
  - definition of error bars as s.d. or s.e.m.

Any descriptions too long for the figure legend should be included in the methods section and/or with the source data.

**In the pink boxes below, please ensure that the answers to the following questions are reported in the manuscript itself. Every question should be answered. If the question is not relevant to your research, please write NA (non applicable). We encourage you to include a specific subsection in the methods section for statistics, reagents, animal models and human subjects.**

### USEFUL LINKS FOR COMPLETING THIS FORM

http://www.antibodypedia.com
http://1degreebio.org
http://www.equator-network.org/reporting-guidelines/improving-bioscience-research-repo
http://grants.nih.gov/grants/olaw/olaw.htm
http://www.mrc.ac.uk/Ourresearch/Ethicsresearchguidance/Useofanimals/index.htm
http://ClinicalTrials.gov
http://www.consort-statement.org
http://www.consort-statement.org/checklists/view/32-consort/66-title
http://www.equator-network.org/reporting-guidelines/reporting-recommendations-for-tum
http://datadryad.org
http://figshare.com
http://www.ncbi.nlm.nih.gov/gap
http://www.ebi.ac.uk/ega
http://biomodels.net/
http://biomodels.net/miriam/
http://jjj.biochem.sun.ac.za
http://oba.od.nih.gov/biosecurity/biosecurity_documents.html
http://www.selectagents.gov/

### B- Statistics and general methods

Please fill out these boxes ➔ (Do not worry if you cannot see all your text once you press return)

| Question | Answer |
|---|---|
| 1.a. How was the sample size chosen to ensure adequate power to detect a pre-specified effect size? | Based on experience, sample sizes between N=10 - 50 do not show significant differences in the range of within-sample-variability, but reliably between independently confirmable genotypes |
| 1.b. For animal studies, include a statement about sample size estimate even if no statistical methods were used. | NA |
| 2. Describe inclusion/exclusion criteria if samples or animals were excluded from the analysis. Were the criteria pre-established? | NA |
| 3. Were any steps taken to minimize the effects of subjective bias when allocating animals/samples to treatment (e.g. randomization procedure)? If yes, please describe. | No |
| For animal studies, include a statement about randomization even if no randomization was used. | NA |
| 4.a. Were any steps taken to minimize the effects of subjective bias during group allocation or/and when assessing results (e.g. blinding of the investigator)? If yes please describe. | No |
| 4.b. For animal studies, include a statement about blinding even if no blinding was done | NA |
| 5. For every figure, are statistical tests justified as appropriate? | Yes |
| Do the data meet the assumptions of the tests (e.g., normal distribution)? Describe any methods used to assess it. | Since most of our data are non-parametric (not continous) we routinely use the unpaired Wilcoxon test, e. g., for quantification of the severity of pruning defects. For the assessment of unpruned neurons (yes or no) we used the  Fisher's exact test. |

| | |
|---|---|
| Is there an estimate of variation within each group of data? | No, does not apply for Wilcoxon's test |
| Is the variance similar between the groups that are being statistically compared? | usually yes but not always (e. g., due to differing penetrance of mutant or RNAi phenotypes) |

### C- Reagents

| | |
|---|---|
| 6. To show that antibodies were profiled for use in the system under study (assay and species), provide a citation, catalog number and/or clone number, supplementary information or reference to an antibody validation profile. e.g., Antibodypedia (see link list at top right), 1DegreeBio (see link list at top right). | |
| 7. Identify the source of cell lines and report if they were recently authenticated (e.g., by STR profiling) and tested for mycoplasma contamination. | NA |

* for all hyperlinks, please see the table at the top right of the document

### D- Animal Models

| | |
|---|---|
| 8. Report species, strain, gender, age of animals and genetic modification status where applicable. Please detail housing and husbandry conditions and the source of animals. | Drosophila melanogaster, third instar larval stage, white pupal stage (0 h APF), 2h APF, 6h APF, 18h APF |
| 9. For experiments involving live vertebrates, include a statement of compliance with ethical regulations and identify the committee(s) approving the experiments. | NA |
| 10. We recommend consulting the ARRIVE guidelines (see link list at top right) (PLoS Biol. 8(6), e1000412, 2010) to ensure that other relevant aspects of animal studies are adequately reported. See author guidelines, under 'Reporting Guidelines'. See also: NIH (see link list at top right) and MRC (see link list at top right) recommendations. Please confirm compliance. | NA |

### E- Human Subjects

| | |
|---|---|
| 11. Identify the committee(s) approving the study protocol. | NA |
| 12. Include a statement confirming that informed consent was obtained from all subjects and that the experiments conformed to the principles set out in the WMA Declaration of Helsinki and the Department of Health and Human Services Belmont Report. | NA |
| 13. For publication of patient photos, include a statement confirming that consent to publish was obtained. | NA |
| 14. Report any restrictions on the availability (and/or on the use) of human data or samples. | NA |
| 15. Report the clinical trial registration number (at ClinicalTrials.gov or equivalent), where applicable. | NA |
| 16. For phase II and III randomized controlled trials, please refer to the CONSORT flow diagram (see link list at top right) and submit the CONSORT checklist (see link list at top right) with your submission. See author guidelines, under 'Reporting Guidelines'. Please confirm you have submitted this list. | NA |
| 17. For tumor marker prognostic studies, we recommend that you follow the REMARK reporting guidelines (see link list at top right). See author guidelines, under 'Reporting Guidelines'. Please confirm you have followed these guidelines. | NA |

### F- Data Accessibility

| | |
|---|---|
| 18: Provide a "Data Availability" section at the end of the Materials & Methods, listing the accession codes for data generated in this study and deposited in a public database (e.g. RNA-Seq data: Gene Expression Omnibus GSE39462, Proteomics data: PRIDE PXD000208 etc.) Please refer to our author guidelines for 'Data Deposition'.<br><br>Data deposition in a public repository is mandatory for:<br>a. Protein, DNA and RNA sequences<br>b. Macromolecular structures<br>c. Crystallographic data for small molecules<br>d. Functional genomics data<br>e. Proteomics and molecular interactions | NA |
| 19. Deposition is strongly recommended for any datasets that are central and integral to the study; please consider the journal's data policy. If no structured public repository exists for a given data type, we encourage the provision of datasets in the manuscript as a Supplementary Document (see author guidelines under 'Expanded View' or in unstructured repositories such as Dryad (see link list at top right) or Figshare (see link list at top right). | NA |
| 20. Access to human clinical and genomic datasets should be provided with as few restrictions as possible while respecting ethical obligations to the patients and relevant medical and legal issues. If practically possible and compatible with the individual consent agreement used in the study, such data should be deposited in one of the major public access-controlled repositories such as dbGAP (see link list at top right) or EGA (see link list at top right). | NA |
| 21. Computational models that are central and integral to a study should be shared without restrictions and provided in a machine-readable form. The relevant accession numbers or links should be provided. When possible, standardized format (SBML, CellML) should be used instead of scripts (e.g. MATLAB). Authors are strongly encouraged to follow the MIRIAM guidelines (see link list at top right) and deposit their model in a public database such as Biomodels (see link list at top right) or JWS Online (see link list at top right). If computer source code is provided with the paper, it should be deposited in a public repository or included in supplementary information. | NA |

### G- Dual use research of concern

| | |
|---|---|
| 22. Could your study fall under dual use research restrictions? Please check biosecurity documents (see link list at top right) and list of select agents and toxins (APHIS/CDC) (see link list at top right). According to our biosecurity guidelines, provide a statement only if it could. | NA |
